# Organization of the pronephric kidney revealed by large-scale gene expression mapping

**DOI:** 10.1186/gb-2008-9-5-r84

**Published:** 2008-05-20

**Authors:** Daniela Raciti, Luca Reggiani, Lars Geffers, Qiuhong Jiang, Francesca Bacchion, Astrid E Subrizi, Dave Clements, Christopher Tindal, Duncan R Davidson, Brigitte Kaissling, André W Brändli

**Affiliations:** 1 Institute of Pharmaceutical Sciences, Department of Chemistry and Applied Biosciences, ETH Zürich, Wolfgang-Pauli-Strasse 10, CH-8093 Zürich, Switzerland; 2 Department of Genes and Behavior, Max Planck Institute for Biophysical Chemistry, Am Fassberg 11, D-37077 Göttingen, Germany; 3 MRC Human Genetics Unit, Western General Hospital, Crewe Road, Edinburgh EH4 2XU, UK; 4 Institute of Anatomy, University of Zürich, Winterthurerstrasse 190, CH-8057 Zürich, Switzerland

## Abstract

**Background:**

The pronephros, the simplest form of a vertebrate excretory organ, has recently become an important model of vertebrate kidney organogenesis. Here, we elucidated the nephron organization of the *Xenopus *pronephros and determined the similarities in segmentation with the metanephros, the adult kidney of mammals.

**Results:**

We performed large-scale gene expression mapping of terminal differentiation markers to identify gene expression patterns that define distinct domains of the pronephric kidney. We analyzed the expression of over 240 genes, which included members of the solute carrier, claudin, and aquaporin gene families, as well as selected ion channels. The obtained expression patterns were deposited in the searchable European Renal Genome Project *Xenopus *Gene Expression Database. We found that 112 genes exhibited highly regionalized expression patterns that were adequate to define the segmental organization of the pronephric nephron. Eight functionally distinct domains were discovered that shared significant analogies in gene expression with the mammalian metanephric nephron. We therefore propose a new nomenclature, which is in line with the mammalian one. The *Xenopus *pronephric nephron is composed of four basic domains: proximal tubule, intermediate tubule, distal tubule, and connecting tubule. Each tubule may be further subdivided into distinct segments. Finally, we also provide compelling evidence that the expression of key genes underlying inherited renal diseases in humans has been evolutionarily conserved down to the level of the pronephric kidney.

**Conclusion:**

The present study validates the *Xenopus *pronephros as a genuine model that may be used to elucidate the molecular basis of nephron segmentation and human renal disease.

## Abstract 2

Gene expression mapping reveals 8 functionally distinct domains in the Xenopus pronephros. Interestingly, no structure equivalent to the mammalian collecting duct is identified.

## Background

The kidney plays a pivotal role in fluid filtration, absorption and excretion of solutes, and in maintaining chemical homeostasis of blood plasma and intercellular fluids. Its primary architectural unit is the nephron, which is a complex structure composed of at least 12 segments that differ in both cellular anatomy and function [1-3]. Each nephron segment is composed of one or more highly specialized cell types that exhibit different patterns of gene expression and, in some cases, even have different embryological origins [4]. In humans, there are about 1 million nephrons per kidney [5]. Each nephron is composed of a filtering component (the renal corpuscle) and a tubule (the renal tubule). Along the tubular portion of the mammalian nephron, four main compartments have been identified: proximal tubule, intermediate tubule, distal tubule, and collecting duct. These four structures can be further subdivided into separate segments based on histological criteria [2,3]. Each nephron segment fulfills distinct physiological functions. The proximal tubules, for instance, return much of the filtrate to the blood circulation in the peritubular capillaries by actively transporting small molecules from the tubular lumen across renal epithelia to the interstitial space, whereas the collecting duct system plays a major role in regulating acid-base balance and urine volume [6,7].

Segmentation of the developing nephron is a crucial step for successful kidney organogenesis. Much of our knowledge of kidney development is focused on the initial stages of kidney formation, where we have gained major insights into the transcription factors and signaling pathways that regulate the induction of nephrogenesis [8,9]. In contrast, little is known about how distinct segments arise along the proximodistal axis of the nascent nephron. Vertebrate kidneys are derived from the intermediate mesoderm in a process that involves inductive interactions, mesenchyme-to-epithelium transitions, and branching morphogenesis to generate the number of nephrons appropriate for the kidney type [4,10]. Three different kidney forms - the pronephros, the mesonephros, and the metanephros - arise sequentially during vertebrate embryogenesis. Although each kidney form differs in overall organization and complexity, they all have the nephron as their basic structural and functional unit. The pronephros is the embryonic kidney of fish and amphibians, in which its function is essential for the survival of the larvae [11]. Because of its anatomical simplicity, the pronephros has recently emerged as an attractive model in which to study human kidney development and disease [12,13].

In *Xenopus*, the pronephric kidneys form as bilateral excretory organs consisting of single nephrons [14,15]. From a structural point of view, the pronephric kidney was thought to be composed of three basic components [14,15]: the glomerulus (or glomus), which is the site of blood filtration; the tubules, where reabsorption of solutes occurs; and the duct, which conveys the resulting urine to the cloaca. Evidence for a more complex nephron organization of the amphibian pronephros was provided by ultrastructural studies [16], and at the molecular level by the regionalized expression of solute transporters and ion channels along the proximodistal axis [17-21]. Based on the expression domains of nine transporter genes, a more refined model of the pronephros consisting of distinct domains and subdomains within the tubules and duct was proposed [19]. To date, however, a comprehensive model of pronephric nephron organization remains elusive. Furthermore, the functional correspondence of the pronephric subdomains to the nephron segments of the mammalian metanephric kidney is poorly understood. We recently proposed a novel model of the *Xenopus *pronephric kidney, which served as a basis for dissecting the roles of *irx *genes in nephron segmentation [22]. In the present study we provide complete molecular evidence supporting our model of the segmental organization of the pronephric nephron, we define the physiological functions associated with each nephron segment, and we reveal the extensive analogies with the mammalian metanephric nephron.

Large-scale gene expression analysis by whole-mount *in situ *hybridization in *Xenopus *embryos has been used successfully in the past to identify new molecular markers and has provided novel insights into the molecular anatomy of embryonic patterning and regionalization [23,24]. Here, we performed a large-scale gene expression screen of the developing pronephros with more than 240 genes encoding terminal differentiation markers to identify previously unappreciated compartments of the mature pronephric kidney in *Xenopus*. Our primary focus was on studying the expression of solute carrier (*slc*) gene family members, which represent - with more than 350 genes - a large portion of the transporter-related genes found in vertebrate genomes [25]. In the mammalian kidney, cohorts of *slc *gene family members are expressed in a segmental manner along the nephron [26].

In the present work, we report the identification of well over 100 *slc *genes with highly regionalized pronephros-specific gene expression patterns in *Xenopus*, suggesting an unprecedented complexity of physiological activities. The obtained gene expression data were organized in an interactive gene expression atlas, which is housed at the European Renal Genome Project (EuReGene) *Xenopus *Gene Expression Database (XGEbase) [27]. Systematic mapping of the gene expression domains revealed the existence of eight molecularly defined segments of the pronephric kidney that are arranged in four distinct tubules along the proximodistal axis of the nephron. By comparative gene expression analysis, we demonstrate remarkable analogies between the tubules of the pronephric and metanephric kidneys. On this basis, we propose a novel model of pronephric kidney organization that emphasizes similarities with the mammalian nephron and uses related nomenclature. Furthermore, we show that genes implicated in human familial renal diseases such as Bartter's syndrome, Gitelman's syndrome, and primary hypomagnesemia are expressed in the corresponding pronephric segments. The pronephric nephron model, together with the collection of more than 100 novel segment-specific marker genes reported here, represents an essential framework with which to dissect the molecular basis of vertebrate nephron segmentation in the *Xenopus *embryo model and may contribute to our understanding of human renal disease.

## Results

### Genome-wide *slc *gene expression analysis defines a large panel of pronephric marker genes

A genome-scale, whole-mount *in situ *hybridization screen was performed to evaluate the expression of solute carrier (*slc*) genes during *Xenopus *pronephric kidney development. We mined public databases to identify cDNAs encoding *Xenopus laevis slc *genes. In total, 225 unique *slc Xenopus *cDNAs were identified that encoded genuine orthologs of human *SLC *genes, based on phylogenetic analyses and synteny mapping (DR and AWB, unpublished data). The retrieved *Xenopus slc *orthologs represent 64% of all human *SLC *genes (total 352).

**Figure 1:**
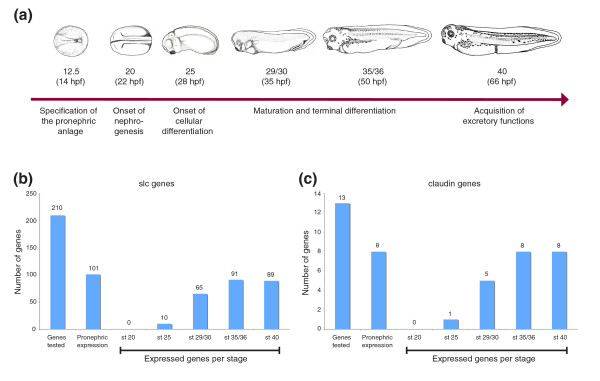
Pronephric kidney development and the global expression of *slc *and *cldn *genes. **(a) **Hallmarks of pronephric kidney development in *Xenopus laevis*. Schematic representations of *Xenopus *embryos are shown with the embryonic stages and hours postfertilization (hpf), in accordance with the terminology established by Nieuwkoop and Faber [28]. Stage 12.5 and 20 embryos are dorsal views with anterior to the left. All other embryos are shown as lateral views. **(b,c) **Complexity of *slc *(panel b) and *cldn *(panel c) gene expression at defined stages of pronephric kidney development. The number of expressed genes at a given stage of pronephric kidney development was determined by whole-mount *in situ *hybridization.

Gene expression patterns were analyzed by whole-mount *in situ *hybridization using *Xenopus *embryos at selected developmental stages, in accordance with the terminology established by Nieuwkoop and Faber (1956) [28]: 20 (22 hours postfertilization [hpf]), 25 (28 hpf), 29/30 (35 hpf), 35/36 (50 hpf), and 40 (66 hpf). The stages were chosen to cover the key steps of pronephric kidney organogenesis: initiation of nephrogenesis (stage 20), onset of cellular differentiation (stage 25), maturation and terminal differentiation (stages 29/30 and 35/36), and acquisition of full excretory organ functions (stage 40; Figure 1a) [14,15].

Of the 225 *slc *genes identified, we detected expression of 210 genes during the embryonic stages tested, and thereof 101 genes (48%) were expressed specifically during pronephric kidney development (Figure 1b). The first evidence for pronephric expression of *slc *genes was identified at stage 25, at which ten genes could be detected (Figure 1b). These included the Na-K-Cl transporter *slc12a1 *(*nkcc2*), the facilitated glucose transporter *slc2a2 *(*glut2*) and the amino acid transporters *slc6a14*, *slc7a3*, and *slc7a7 *(Additional data file 1). By stage 29/30, expression of 65 genes - representing the majority (64%) of the *slc *genes tested - could be detected. This correlates well with the onset of epithelial differentiation and lumen formation [14,15]. The number of expressed *slc *genes increases to 91 and 89 at stages 35/36 and 40, respectively (Figure 1b), as the pronephric nephron undergoes terminal differentiation and acquires characteristics of a functional excretory organ. Complete lists of *slc *genes expressed for each stage of pronephric development tested are provided in Additional data file 1.

### A comprehensive model for pronephric segmentation revealed by slc gene expression mapping

Our gene expression studies indicated that all 101 *slc *genes exhibited spatially restricted expression patterns in the developing pronephric kidney. Because *slc *genes encode terminal differentiation markers, we reasoned that a systematic analysis of the *slc *gene expression domains could reveal the underlying segmental organization of the differentiated pronephric nephron.

The nephron of the stage 35/36 pronephric kidney was selected for *slc *gene expression mapping along the proximodistal axis. Robust expression of most *slc *genes was evident by this stage, which preceded the onset of pronephric functions by about 3 hours. Furthermore, the stage 35/36 nephron retains a simple structure, lacking areas of extensive tubular convolution. It is largely a linear epithelial tube stretched out along the anteroposterior body axis. Characteristic morphological landmarks (somites, thickenings, and looped areas of the nephron) facilitate the mapping of the gene expression domains that can be performed on whole embryos without need for sectioning. A contour map of the stage 35/36 nephron was developed from embryos subjected to whole-mount *in situ *hybridization with *fxyd2*, *pax2*, and *wnt4 *probes (see Materials and methods, below, for details). The obtained model covered the three nephrostomes, which mark the most proximal end of the nephron, followed by three tubules, which merge to form a long-stretched duct that connects at its distal end to the cloaca. Subsequently, the expression domains of each *slc *gene were carefully mapped onto the stage 35/36 model nephron.

**Figure 2:**
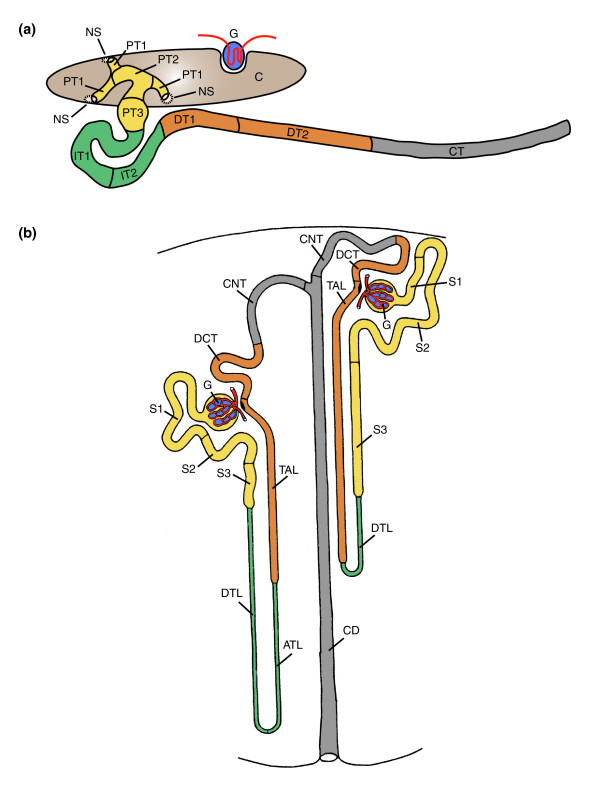
Models of the segmental organization of the *Xenopus *pronephric and mammalian metanephric nephrons. The color coding of analogous nephron segments is based on the comparison of marker gene expression as shown in Figure 7. **(a) **Schematic representation of the stage 35/36 *Xenopus *pronephric kidney. The glomerular filtration apparatus (G; also known as glomus) is derived from the splanchnic layer of the intermediate mesoderm and receives blood from vessels that branch from the dorsal aorta. All other parts of the pronephric nephron are derivatives of the somatic layer of the intermediate mesoderm. On the basis of molecular markers, four distinct tubular compartments can be recognized. Each tubule may be further subdivided into distinct segments: proximal tubule (PT, yellow; PT1, PT2, and PT3), intermediate tubule (IT, green; IT1 and IT2), distal tubule (DT, orange; DT1 and DT2), and connecting tubule (CT, gray). The nephrostomes (NS) are ciliated peritoneal funnels that connect the coelomic cavity (C) to the nephron. The scheme was adapted from Reggiani and coworkers [22]. **(b) **Scheme depicting a short-looped and a long-looped nephron of the adult mammalian metanephric kidney. The figure was taken and adapted from Kriz and Bankir [2]. Abbreviations used for the mammalian nephron segments are as follows: ATL, ascending thin limb; CD, collecting duct; CNT, connecting tubule; DCT, distal convoluted tubule; DTL, descending thin limb; S1, S2, and S3, segments of the proximal tubule; TAL, thick ascending limb.

The segmental organization that emerged from *slc *gene expression mapping is shown in Figure 2a. It revealed a previously unappreciated complexity and extends an older model reported by Zhou and Vize [19]. In addition to the nephrostomes, which connect the pronephric nephron to the coelomic cavity and the glomerular filtration apparatus, eight functionally distinct segments were defined. Cross-species gene expression comparisons were performed to delineate similarities between the *Xenopus *pronephric and mammalian metanephric nephron (see below). These studies revealed striking analogies, allowing us to adopt a nomenclature for the pronephric segments that largely follows the widely accepted one used for the mammalian metanephros [2], which is shown in Figure 2b. The pronephric nephron of *Xenopus *is composed of four basic domains: proximal tubule, intermediate tubule, distal tubule, and connecting tubule. Each tubule may be further subdivided into distinct segments. The proximal tubule (PT) is divided into three segments (PT1, PT2, and PT3), whereas the intermediate tubule (IT) and the distal tubule (DT) are both composed of two segments IT1 and IT2, and DT1 and DT2, respectively. In contrast, the connecting tubule (formerly known as pronephric duct) does not appear to be further subdivided. The molecular evidence supporting the proposed segmentation model and nomenclature are discussed in detail below.

### Distribution of *slc *gene expression in the pronephric kidney

The complete annotation of the pronephric expression domains for each *slc *gene can be found in Additional data file 2. The *slc *gene expression domains were characterized by sharp, conserved expression boundaries, which define the limits of the segments and tubules. A given expression domain could either be confined to a single segment, comprise an entire tubule, or spread over more than one tubule. Of the 91 *slc *genes analyzed for expression in the stage 35/36 pronephric kidney, we detected expression of 75 genes in the proximal tubule, 27 genes in the intermediate tubule, 24 genes in the distal tubule, and 13 genes in the connecting tubule (Additional data files 3 to 6).

### Expression domains of *slc *genes define three segments in the proximal tubule

**Figure 3:**
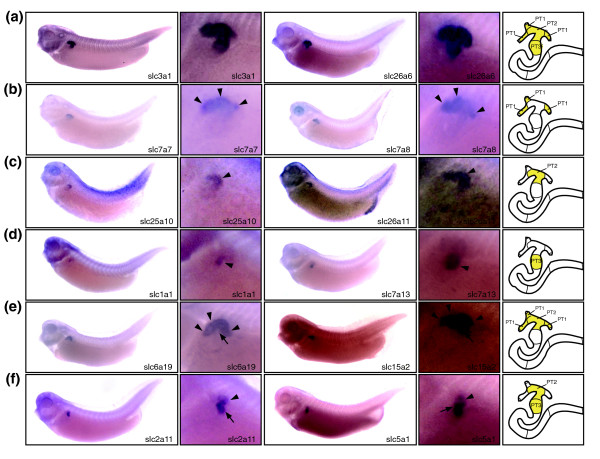
The expression domains of *slc *genes identify three distinct segments in the proximal tubule. Stage 35/36 *Xenopus *embryos were stained for marker gene expression by whole-mount *in situ *hybridization. For each distinct class of expression pattern obtained, lateral views of embryos stained for two representative *slc *genes are shown accompanied by enlargements of the pronephric region. A color-coded scheme of the nephron depicts the deduced segmental expression domains. **(a) **Examples of *slc *genes expressed in all segments of the proximal tubule. **(b-d) **Examples of *slc *genes with expression confined to proximal tubule (PT)1 (panel b), PT2 (panel c), or PT3 (panel d) alone. Arrowheads are shown to highlight specific proximal tubule segments stained. **(e,f) **Examples of *slc *genes with expression either in PT1 and PT2 (panel e) or in PT2 and PT3 (panel f). In panel e, arrowheads and arrows highlight the PT1 and PT2 segments, respectively. In panel f, arrowheads and arrows highlight the PT2 and PT3 segments, respectively. The localization of the *slc7a13 *expression domains has previously been reported [22]. They are shown here for comparative purposes.

With 75 genes, the proximal tubules exhibited the greatest complexity of *slc *gene expression. This underscores their importance in reabsorbing diverse classes of solutes from the glomerular ultrafiltrate. We identified 26 genes with exclusive expression throughout the proximal tubule compartment. Among these, 18 were strongly expressed and included *slc2a2*, *slc3a1*, *slc4a7*, *slc5a11*, *slc22a5*, and *slc26a1 *(Figure 3a and Additional data file 2). The expression domains of other *slc *genes revealed a further subdivision of the proximal tubule into three distinct segments (PT1, PT2, and PT3). This tripartite organization is reminiscent of mammalian proximal tubules, which are commonly subdivided into S1, S2, and S3 segments [2].

Two genes were predominantly expressed in PT1 (the most proximal segment of the proximal tubule), namely *slc7a7 *and *slc7a8*. Low levels of expression could also be detected in PT2 (Figure 3b and Additional data file 2). Interestingly, all three PT1 segments appear to be equivalent, because we do not have evidence for differential expression of marker genes. Two genes, namely *slc25a10 *and *slc26a11*, were exclusively expressed in PT2 (Figure 3c), and *slc1a1 *and *slc7a13 *were confined to PT3 (Figure 3d). Furthermore, we found several examples of *slc *gene expression encompassing two segments. Twelve genes including *slc5a2 *[22], *slc6a19*, and *slc15a2 *were expressed in PT1 as well as PT2 (Figure 3e and Additional data file 2). In contrast, 13 *slc *genes, including *slc2a11 *and *slc5a1*, were detected in both PT2 and PT3 (Figure 3f and Additional data file 2). The molecular subdivision of the proximal tubule revealed by segment-specific markers is also evident morphologically. Three PT1 segments connect the nephrostomes to a single PT2 segment. The adjacent distal region corresponds to PT3 and can be identified as a bulging of the proximal tubule, which is also known as the broad or common tubule [29].

### Expression of *slc *genes delineate the intermediate tubule as a bipartite structure

**Figure 4:**
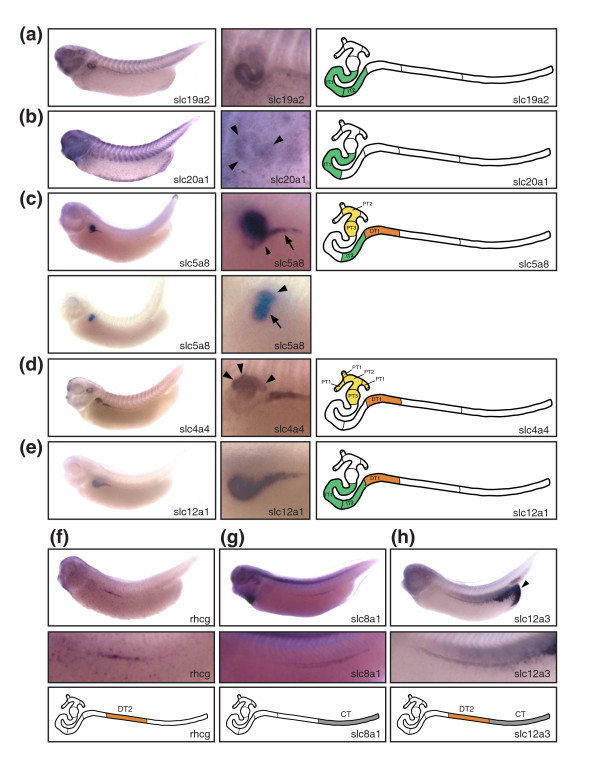
*Slc *gene expression defines segmentation of the intermediate, distal, and connecting tubules. Stage 35/36 *Xenopus *embryos were stained for marker gene expression by whole-mount *in situ *hybridization. Lateral views of stained embryos are shown accompanied by enlargements of the pronephric region and a color-coded scheme of the nephron depicting the deduced segmental expression domains. **(a) ***slc19a2*: intermediate tubule. **(b) ***slc20a1*: intermediate tubule (IT)1 (arrowheads). **(c) ***slc5a8*: proximal tubule (PT)2, PT3, IT2, and distal tubule (DT)1. In the upper panel, the embryo was stained to reveal *slc5a8 *expression in IT2 (arrow) and DT1 (arrowhead). The embryo shown in the lower panel was stained shorter to demonstrate expression in PT2 (arrowhead) and PT3 (arrows). **(d) ***slc4a4*: proximal tubules, DT1. Arrowheads illustrate expression in PT1. **(e) ***slc12a1*: intermediate tubule, DT1. **(f) ***rhcg*/*slc42a3*: DT2. **(g) ***slc8a1*: connecting tubule (CT). **(h) ***slc12a3*: DT2, CT. Note that there is also strong *slc12a3 *expression in the cloaca (arrowhead). The localization of the expression domains for *slc12a1 *and *slc12a3 *has previously been reported [22]. They are shown here for comparative purposes.

The intermediate tubule, an S-shaped structure, follows distal to the proximal tubule in the stage 35/36 pronephric nephron (Figure 2a). It is characterized molecularly by the expression of the thiamine transporter *slc19a2 *(Figure 4a). In addition, *slc *genes with nonexclusive expression in the intermediate tubule include *slc4a11*, *slc12a1*, *slc16a7*, and *slc25a11 *(Figure 4e and Additional data file 2). For example, *slc12a1 *expression extends into the distal tubule to include DT1 (Figure 4e). The boundaries of the intermediate tubule are also defined by *slc4a4*, which was not detected in the intermediate tubule but was prominently expressed in the flanking proximal and distal tubule domains (Figure 4d).

The intermediate tubule is comprised of two segments, namely IT1 and IT2. The molecular evidence for this subdivision was provided by the expression of *slc20a1 *in the proximal part (IT1) and *slc5a8 *in the distal part (IT2; Figure 4b,c). Although *slc5a8 *expression occurs also in the proximal tubules (PT2 and PT3) and in the distal tubule (DT1), the expression domain in the intermediate tubules defines unequivocally the boundary between IT1 and IT2 (Figure 4c). The bipartite nature of the intermediate tubule is further supported by the expression of *irx *transcription factor family members *irx1*, *irx2*, and *irx3 *[22].

### Organization of distal and connecting tubules revealed by *slc *gene expression

The distal tubule occupies roughly the proximal half of the stretch-out part of the pronephric nephron (Figure 2a). To date we have failed to identify an *slc *gene with expression in the entire distal tubule only. However, the distal expression domain of *slc16a6 *comprises the entire distal tubule (Additional data file 2). The distal tubule is composed of two distinct segments: DT1 and DT2. Molecularly, DT1 was defined by the expression of the sodium bicarbonate transporter *slc4a4*; however, this transporter also has a second expression domain in the proximal tubule (Figure 4d). In addition, several *slc *genes were identified that have DT1 as their most distal expression domain. These included *slc4a11*, *slc5a8*, and *slc12a1 *(Figure 4c,e and Additional data file 2). DT2 was demarcated by expression of the ammonia transporter *rhcg*/*slc42a3 *(Figure 4f). Furthermore, *slc12a3 *shared DT2 as its most proximal expression domain (Figure 4h).

The connecting tubule links the pronephric kidney to the rectal diverticulum and the cloaca. Two *slc *genes exhibited exclusive expression in this compartment, namely the sodium/calcium exchanger *slc8a1 *and the zinc transporter *slc30a8 *(Figure 4g and Additional data file 2). To date, we have not obtained any evidence supporting further subdivision of the connecting tubule.

### Validation of the pronephric segmentation model

**Figure 5:**
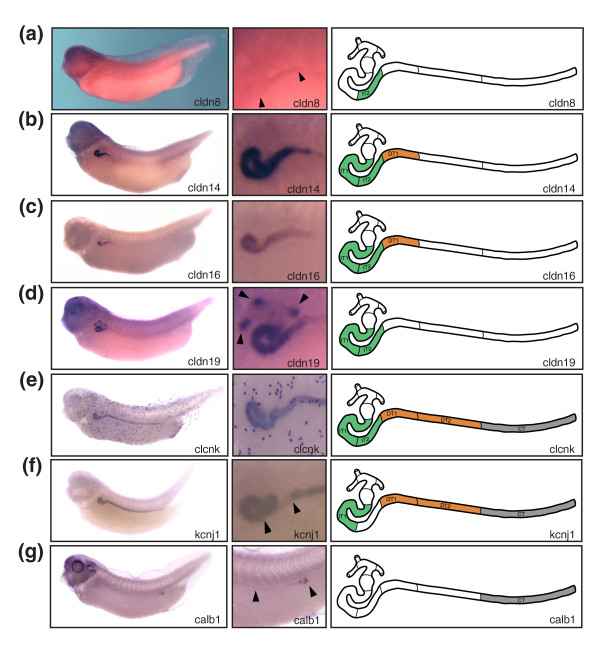
Expression domains of selected molecular marker genes validates the pronephric segmentation model. Whole-mount *in situ *hybridizations of stage 35/36 *Xenopus *embryos were performed. Lateral views of whole embryos (left panels), enlargements of the pronephric region (middle panels), and color-coded schematic representations of the segment-restricted expression domains (right panels) are shown. **(a) ***cldn8*: intermediate tubule (IT)2. Note that the expression levels are low. Arrowheads indicate the proximal and distal boundaries of the *cldn8 *expression domain. **(b,d) ***cldn14 *and *cldn16*: intermediate tubule, distal tubule (DT)1. **(d) ***cldn19*: nephrostomes (arrowheads), intermediate tubule. **(e) ***clcnk*: intermediate tubule, distal tubule, connecting tubule. Note that the dotted staining pattern localizes to cells of the epidermis. The localization of the *clcnk *expression domains has previously been reported [22] and is shown here for comparative purposes. **(f) ***kcnj1*: IT1, distal tubule, connecting tubule. The arrowhead indicates the location of IT2, which fails to express *kcnj1*. **(g) ***calb1*: connecting tubule. The arrowhead indicates the proximal boundary of the expression domain. Expression is highest in the most distal parts of the connecting tubule.

We extended our gene expression analysis to the claudin (*cldn*) gene family and selected other genes to validate the proposed model of pronephric segmentation. Claudins are key components of epithelial tight junctions, where they are responsible for the selectivity and regulation of paracellular permeability [30,31]. In the mammalian kidney, several claudin genes are expressed in segment-specific patterns along the nephron [30,32]. We profiled the claudin gene family for evidence of nephron segment-specific gene expression in *Xenopus*. We retrieved 14 distinct *Xenopus *claudin cDNAs from database searches, which covers 64% of the complement of 22 claudin genes typically found in vertebrate genomes. We analyzed the expression of 13 claudin genes by whole-mount *in situ *hybridization and found that eight genes were expressed in the developing pronephric kidney (Figure 1c and Additional data file 2). No pronephric expression of claudin genes was detected at stage 20. Induction of *cldn6 *expression occurred at stage 25, and by stage 35/36 all eight *cldn *genes were expressed (Figure 1c and Additional data file 1). The temporal profile of claudin gene expression during pronephric kidney development therefore mirrors the situation reported for the *slc *genes (Figure 1b,c). Four *cldn *genes (*cldn3*, *cldn4*, *cldn6*, and *cldn12*) were expressed throughout the entire stage 35/36 nephron. In contrast, expression of the other *cldn *genes was highly regionalized. Interestingly, all shared expression in the intermediate tubule. The *cldn8 *gene had the most restricted expression, being present only in the IT2 segment (Figure 5a). Apart from the intermediate tubule, the expression domains of *cldn14 *and *cldn16 *extended distally to include DT1 (Figure 5b,c). Finally, transcripts for *cldn19 *were present not only in the intermediate tubule but also in the nephrostomes (Figure 5d).

We also studied the expression of the kidney-specific chloride channel *clcnk*, the potassium channel *kcnj1 *(also known as *romk*), and the calcium-binding protein calbindin 1 (calbindin 28 kDa; *calb1*). Previously, we reported *clcnk *to be a marker of the pronephric duct [17], and more recently mapped its expression to cover the intermediate, distal, and connecting tubules [22] (Figure 5e). Expression of *kcnj1 *was similar to that of *clcnk*, with the exception that *kcnj1 *was not present in IT2 (Figure 5f). Finally, *calb1 *expression was restricted to the connecting tubule with highest expression at the distal tip (Figure 5g). Expression throughout the connecting tubule segment became more apparent by stage 40 (data not shown). In summary, the analysis of additional pronephric marker genes fully supports our proposed model of pronephric nephron segmentation. For example, *cldn8 *and *kcnj1 *expression provides further evidence for the bipartite nature of the intermediate tubule compartment. Furthermore, we failed to detect any evidence for additional subdivisions of the nephron other than the ones reported here.

### Gene expression comparisons reveal striking analogies of nephron segmentation between pronephric and metanephric kidneys

**Figure 6:**
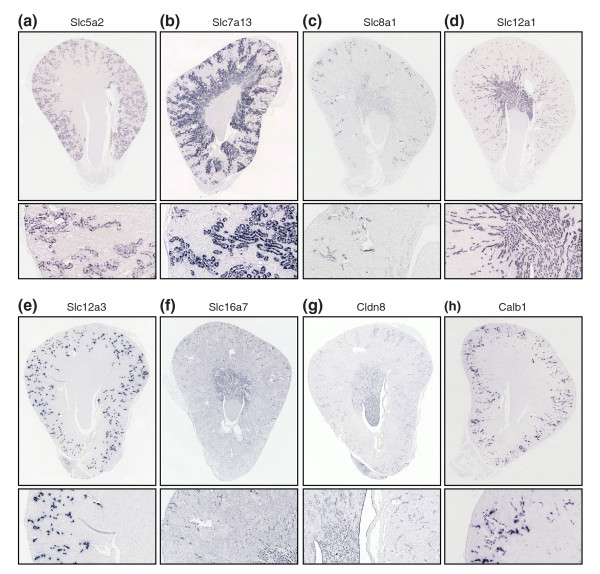
Expression of selected renal marker genes in the adult mouse kidney. *In situ *hybridizations were performed on paraffin sections of adult kidneys taken from 12-week old mice. Whole transverse sections (upper panels) and magnifications (lower panels) are shown to illustrate marker gene expression in detail. **(a) ***Slc5a2*: proximal tubules (S1, S2). **(b) ***Slc7a13*: proximal tubules (S2, S3). **(c) ***Slc8a1*: connecting tubule. **(d) ***Slc12a1*: thick ascending limb. **(e) ***Slc12a3*: distal convoluted tubule. **(f) ***Slc16a7*: thick ascending limb, connecting tubule. **(g) ***Cldn8*: descending thin limb, connecting tubule, collecting duct. **(h) ***Calb1*: distal convoluted tubule, connecting tubule.

We performed cross-species gene expression comparisons to identify similarities between the nephron organization of the *Xenopus *pronephros and the mammalian metanephros. We selected 23 marker genes with highly regionalized expression in the *Xenopus *pronephric kidney to compare their renal expression domains with the corresponding mammalian orthologs. As shown in Table 1, the list included 18 *slc *genes, *calb1*, *cldn8*, *cldn16*, *clcnk*, and *kcnj1*. Information on the expression of the mammalian counterparts in either the adult mouse or rat kidney was obtained in part from the published literature (Table 2). In addition, we determined independently the expression patterns for many of the selected genes by *in situ *hybridization analysis. Selected examples of stained adult mouse kidney sections are shown in Figure 6. We determined the previously unknown renal expression domains of *Slc5a9*, *Slc6a13*, *Slc13a3*, and *Slc16a7 *(Figure 6 and data not shown). Furthermore, we confirmed the expression domains of many others, including *Slc5a2*, *Slc7a13*, *Slc8a1*, *Slc12a1*, *Slc12a3*, *Cldn8*, and *Calb1 *(Table 2, Figure 6, and data not shown).

**Table 1:** Selected marker genes of the stage 35/36 *Xenopus *pronephric nephron

Gene	GenBank accession number	Expression domains	References
*slc1a1*	CV079713	PT3	This study
*slc3a1*	BU903456	PT1, PT2, PT3	This study
*slc4a4*	BU905206	PT1, PT2>PT3; DT1	[19] and this study
*slc5a1*	CA974591	PT2<PT3	This study
*slc5a2*	CF520680	PT1, PT2	[22] and this study
*slc5a9*	CA788193	PT2, PT3	This study
*slc6a13*	BC060418	PT1, PT2, PT3	This study
*slc6a19*	BC081075	PT1, PT2	This study
*slc7a13*	BC060020	PT3	[22] and this study
*slc8a1*	BG371210	CT	This study
*slc12a1*	BU904428	IT1, IT2, DT1	[19,22] and this study
*slc12a3*	CA790325	DT2, CT	[22] and this study
*slc13a3*	BC075138	PT2, PT3	This study
*slc16a7*	BJ059209	IT1, IT2, DT1, DT2, CT	This study
*slc22a2*	BC061664	PT2*, PT3*	This study
*slc22a5*	BC056014	PT1, PT2, PT3	This study
*slc22a6*	BC081057	PT2, PT3	This study
*rhcg/slc42a3*	BC084943	DT2	This study
*cldn8*	DR877133	IT2	This study
*cldn16*	CD100665	IT1, IT2, DT1	This study
*calb1*	U76636	CT	This study
*clcnk*	AJ011385	IT1, IT2, DT1, DT2, CT	[17,22] and this study
*kcnj1*	CF522101	IT1, DT1, DT2, CT	This study

*Expression domains determined for stage 40. CT, connecting tubule; DT, distal tubule; IT, intermediate tubule; PT, proximal tubule;

**Table 2:** Selected marker genes of the adult rodent metanephric nephron

Gene	GenBank accession number	Expression domains	References
*Slc1a1*	NM_009199	S2, S3	[69]
*Slc3a1*	NM_009205	S1, S2, S3	[70] and this study (data not shown)
*Slc4a4*	NM_018760	S1, S2>S3	[71,72] and this study (data not shown)
*Slc5a1*	NM_019810	S2<S3	[73,74]
*Slc5a2*	NM_133254	S1, S2	[32] and this study
*Slc5a9*	NM_145551	S1<S2, S3	This study (data not shown)
*Slc6a13*	NM_144512	S2<S3	This study (data not shown)
*Slc6a19*	NM_028878	S1, S2>S3	[75]
*Slc7a13*	NM_028746	S2, S3	[76] and this study
*Slc8a1*	NM_011406	CNT	[77,78] and this study
*Slc12a1*	NM_183354	TAL	[79] and this study
*Slc12a3*	NM_019415	DCT	[77,80] and this study
*Slc13a3*	NM_054055	S2, S3	This study (data not shown)
*Slc16a7*	NM_011391	TAL, CNT	This study
*Slc22a2*	NM_013667	S2, S3	[81] and this study (data not shown)
*Slc22a5*	NM_011396	S1, S2, S3	[82,83] and this study (data not shown)
*Slc22a6*	NM_008766	S1<S2, S3	[84,85]
*Rhcg/Slc42a3*	NM_019799	DCT, CNT, CD	[51,52]
*Cldn8*	NM_018778	DTL, CNT, CD	[86] and this study
*Cldn16*	NM_053241	TAL	[87]
*Calb1*	NM_009788	DCT, CNT	[80] and this study
*Clcnka*	NM_024412	ATL	[88]
*Clcnkb*	NM_019701	TAL, DCT, CNT, CD	[88]
*Kcnj1*	NM_019659	TAL, DCT, CNT	[89]

ATL, ascending thin limb; CD, collecting duct; CNT, connecting tubule; DCT, distal convoluted tubule; TAL, thick ascending limb.

**Figure 7:**
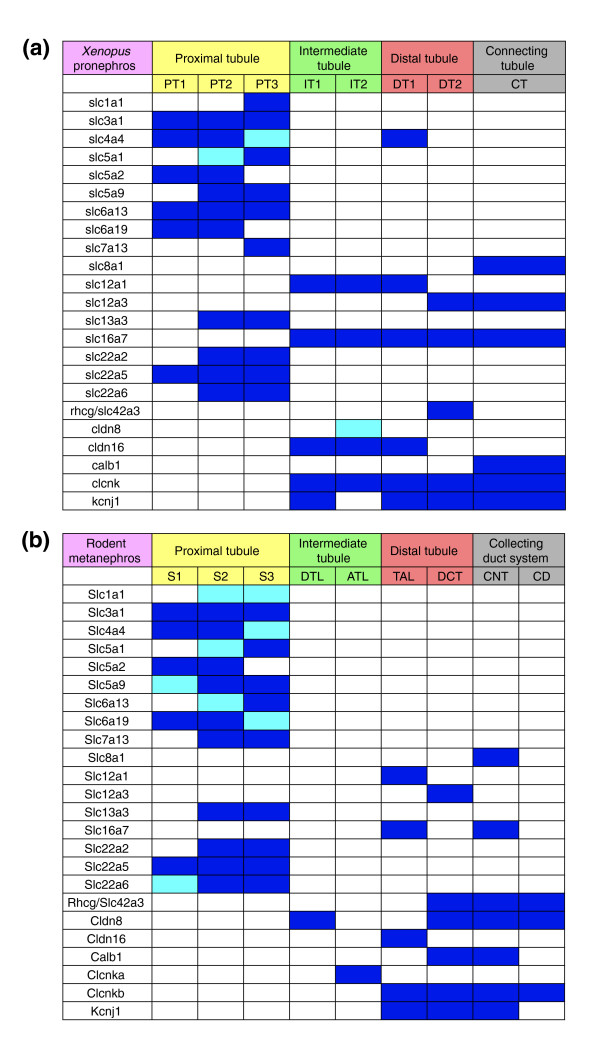
Expression domains of selected marker genes in the *Xenopus *pronephros and the rodent metanephros. The expression domains of selected marker genes in the nephrons of **(a) ***Xenopus *stage 35/36 pronephric kidneys and **(b) **adult rodent metanephric kidneys are depicted schematically. The nephrostomes, which may correspond to the neck segment found in some mammalian species such as rabbits [67,68], are not shown. Dark and pale blue colors indicate strong and low expression levels, respectively. Note that only a single *clcnk *gene is known in *Xenopus*, whereas there are two mouse *Clcnk *genes. The expression of *Xenopus clcnk *in the intermediate, distal, and connecting tubules has therefore to be compared with the combined renal expression domains of mouse *Clcnka *and *Clcnkb*. The abbreviations for segments of the pronephric and metanephric nephrons are given in the legend to Figure 2.

A comparison of the expression domains of the selected marker genes between the *Xenopus *pronephros and the rodent metanephros is shown schematically in Figure 7. Overall, a remarkable conservation of segmental gene expression was observed. This was most striking for the proximal tubule. All 13 mammalian genes with expression in the proximal tubule were also expressed in the *Xenopus *proximal tubule. Generally, only minor differences between *Xenopus *and mammalian marker genes were observed. In many cases, however, we found complete conservation of segmental expression domains. This is best illustrated by the low-affinity and high-affinity Na-glucose transporters *Slc5a2 *and *Slc5a1*, which are sequentially expressed along the proximodistal axis of the proximal tubule [33]. *Slc5a2 *localizes to S1 and S2 in mouse and to PT1 and PT2 in *Xenopus*, whereas *Slc5a1 *was detected in S2 and S3, and PT2 and PT3, respectively (Figure 3e,f, Figure 6a, and data not shown).

The comparison of gene expression in the intermediate tubule revealed a more complex picture. Importantly, there was clear evidence for expression of *Cldn8 *and *Clcnk *in the intermediate tubules of *Xenopus *and mouse. The *Cldn8 *gene, which in mouse is expressed in the descending thin limb, was confined to IT2 in *Xenopus *(Figure 5a and Figure 6g). With regard to *Clcnk*, the broad expression domain (IT1 → connecting tubule) of the single *Xenopus clcnk *gene was comparable to the combined expression domains of *Clcnka *(ascending thin limb) and *Clcnkb *(thick ascending limb [TAL] to collecting duct) in mouse kidney (Figure 5e and data not shown). Moreover, we observed that the *Xenopus *intermediate tubule shares some transport properties with the mammalian TAL. In *Xenopus*, *slc12a1*, *slc16a7*, *cldn16*, and *kcnj1 *- whose murine counterparts are markers of the TAL (Table 2) - exhibited striking proximal expansions of their expression domains to include segments of the intermediate tubule (Figure 7).

The distal tubule in mammals can be divided structurally into two compartments: the TAL and the distal convoluted tubule (DCT). Molecularly, it is defined by the differential expression of the Na-K-Cl transporter *Slc12a1 *in the TAL and the Na-Cl cotransporter *Slc12a3 *in the DCT (Figure 6d,e). We found that this was also the case for the *Xenopus *distal tubule. Note that the junctions between the *slc12a1 *and *slc12a3 *expression domains define the boundary between DT1 and DT2 (Figure 4e,h). We also noticed that the *Xenopus *orthologs of mouse TAL markers were expressed in the *Xenopus *DT1. When comparing the mouse DCT with the *Xenopus *DT2, striking similarities became apparent. As mentioned above, we could demonstrate expressions of the key marker *slc12a3 *and of *clcnk*, *kcnj1*, and the ammonia transporter *Rhcg*/*Slc42a3 *in DT2. However, we found one exception relating to the expression of the calcium-binding protein encoded by *Calb1*, which is a marker of the mouse DCT and connecting tubule (Figure 6h). In *Xenopus*, *calb1 *was not expressed in DT2 but in the connecting tubule only (Figure 5g). Taken together, the *Xenopus *DT1 and DT2 are analogous to the mouse TAL and DCT, respectively.

In mouse kidney, the connecting tubule can be identified on the basis of *Slc8a1 *expression (Figure 6c). Interestingly, expression of *Xenopus slc8a1 *also defines a distinct compartment adjacent to the distal tubule, which we termed connecting tubule. As in the mouse, the *Xenopu*s connecting tubule expresses *slc16a7*, *calb1*, *clcnk*, and *kcnj1*, which further emphasize the similarities between the connecting tubules of the pronephros and metanephros (Figure 5e-g, Figure 6f,h, and data not shown).

### The *Xenopus *pronephric kidney lacks a nephron segment analogous to the mammalian collecting duct

We assessed *Xenopus *embryos for the expression of marker genes of the mammalian collecting duct. First, we analyzed three aquaporin genes, namely *aqp2*, *aqp3*, and *aqp4*, which are markers of principal cells in the mammalian collecting duct [34-37]. None of the tested genes were expressed in the pronephric kidney (data not shown). Subsequently, we analyzed *slc4a1 *(AE1), a marker of type A intercalated cells of the cortical and medullary collecting ducts [38], and *slc26a4 *(pendrin), which is expressed in type B intercalated cells of the connecting tubule and cortical collecting duct in the adult mammalian kidney [39]. No expression could be detected in the stage 35/36 pronephric kidney (data not shown). We therefore conclude that there is presently no molecular evidence indicating that the stage 35/36 *Xenopus *pronephric kidney harbors a nephron segment that shares molecular characteristics with the mammalian collecting duct.

### A public resource: XGEbase

The present study has generated a large, unique dataset of temporal and spatial gene expression patterns. We organized these data online as a public resource in EuReGene in the form of an interactive database. XGEbase [27] currently contains whole-mount *in situ *hybridization data on 210 *slc *genes. The embryonic expression patterns are documented by more than 1,200 representative microscopic *in situ *hybridization images. The pronephric expression patterns are fully annotated in accordance with our model. Moreover, we also identified more than 100 genes expressed in spatially restricted patterns within other non-renal tissues such as brain, liver, and heart (DR and AWB, unpublished data). OK! Although not the explicit focus of the present study, the obtained expression patterns were fully annotated in accordance with the *Xenopus *Anatomy Ontology [40] and deposited in XGEbase. Hence, XGEbase provides not only a unique resource for future studies on pronephric kidney development and function, but also enhances our general understanding of organogenesis in the *Xenopus *model.

## Discussion

### Complexity of the *Xenopus *pronephric kidney revealed by large-scale gene expression mapping

**Figure 8:**
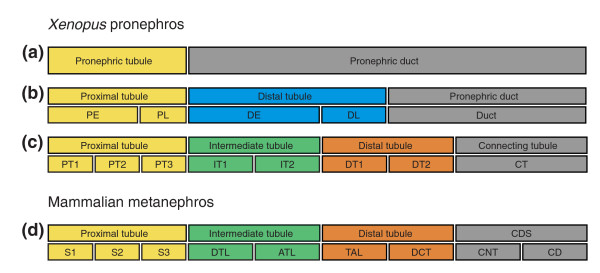
Comparison of vertebrate nephron segmentation models. Schematic representations of **(a) **the original model, **(b) **the improved model of Zhou and Vize [19], and **(c) **our novel model of pronephric nephron segmentation in the stage 35/36 *Xenopus *embryo. For comparison, **(d) **a simplified scheme of the model of the segmental organization of the mammalian metanephric nephron, according to Kriz and Bankir [2], is shown. The glomerulus and nephrostomes (neck segments) are not shown. Abbreviations: DE, distal early; DL, distal late; PE, proximal early; PL, proximal late. For other abbreviations, see the legends to Figure 2.

The pronephric nephron was until recently considered to be a simple structure composed of pronephric tubules and the pronephric duct [14,15] (Figure 8a). This apparent simplicity of the bilateral excretory organs of amphibian larvae was first recognized almost 180 years ago with the identification of the Wolffian body (*Wolffschen Körper*) and the associated excretory duct (*Ausführungsgang*) [41]. A more complex view emerged from a reconstruction of the larval pronephros of *Bufo viridis *combined with ultrastructural examinations [16]. Excluding the nephrostomes, the pronephric nephron was subdivided into three domains (Figure 8b): a proximal tubule composed of columnar epithelia with well developed brush borders, followed by a distal tubule with cuboidal epithelia, and a duct with low cylindrical epithelial cells. Based on the localized expression of nine membrane transporter genes, additional early and late subdomains of both the proximal and distal tubules were identified molecularly, and a terminology was adopted that was previously used for the mesonephric nephron [19] (Figure 8b). The degree of similarity between the nephron organization of the *Xenopus *pronephros and the mammalian metanephros remains unclear, however.

Our analysis, which included more than 100 molecular marker genes, revealed an even more complex picture. Although we were able to confirm the compartment boundaries defined by Zhou and Vize [19], our analysis revealed three additional domains of the pronephric nephron, which had not previously been described. Further subdivisions of the early proximal tubule, the early distal tubule, and duct were recognized, culminating in the most comprehensive model of pronephric nephron segmentation reported to date (Figure 8c).

### An evolutionary perspective on vertebrate nephron organization

Similarities between the eight distinct *Xenopus *pronephric nephron segments and mammalian metanephric nephron segments were established on the basis of conserved marker gene expression (Figure 7). The basic architecture of the mammalian nephron - with four main compartments (the proximal tubule, intermediate tubule, distal tubule, and collecting duct system) and their further subdivision - are well established on the basis of morphological criteria [2,3] (Figure 2b and Figure 8d). Remarkably, the expression of many marker genes in the nephron segments of the mammalian metanephros was confined to equivalent segments of the *Xenopus *pronephros. On the basis of these findings, we have redefined the molecular anatomy of the *Xenopus *pronephros and propose a novel nomenclature that acknowledges the striking similarities with the mammalian nephron (Figure 8c). Unlike previous models of the zebrafish and *Xenopus *pronephros [16,19,42], we define an intermediate tubule compartment, identify a segment with characteristics of the distal convoluted tubule, and clarify the analogies between the pronephric duct and the mammalian collecting duct system. Our findings suggest that the basic architecture of the nephron evolved early in vertebrate evolution and that the last common ancestor of mammals and amphibians, more than 360 million years ago [43,44], must have already possessed excretory organs comprised of four distinct, segmented tubules. Clearly, subsequent evolution has modified this basic architecture in a species-specific manner to meet the differing physiological requirements of vertebrates residing in diverse sets of habitats and environments.

### The pronephric proximal tubule shares many transport activities with its metanephric counterpart

By focusing the large-scale gene expression analysis of the pronephric kidney on *slc *genes, we have now obtained unprecedented insights into the diversity and scope of physiological transport activities carried out by the pronephric kidney. The panel of 225 *slc *genes included representatives of 46 *slc *gene families. We found that *slc *genes representing 35 *slc *gene families were clearly expressed in the pronephric kidney. Remarkably, of the more than 100 *slc *genes with pronephric expression we had identified, 75 were expressed in the proximal tubule.

The mammalian proximal tubule is responsible for bulk reabsorption of more than 70% of the filtered solutes in the primary urine, which includes ions (sodium, chloride, potassium, calcium, phosphate, and bicarbonate), vital nutrients (glucose and amino acids), and water. Molecular evidence that the proximal domains of the pronephric kidney can support some of these transport activities was reported previously [17,19,20,45]. The present study uncovers, on the basis of *slc *gene expression patterns, the broad scope of inferred transport activities carried out by pronephric proximal tubules (Additional data file 3). We provide here evidence for the expression of transporters that mediate uptake of glucose (members of the *slc2 *and *scl5 *gene families), amino acids (*slc1*, *slc3*, *slc7*, *slc17*, *slc36*, and *slc38*), peptides (*slc15*), bicarbonate (*slc4*), acetyl-coenzyme A (*slc33*), nucleosides (*slc28 *and *slc29*), vitamins (*slc19 *and *slc23*), and metal ions (*slc30*, *slc31*, and *slc39*). Apart from reabsorptive activities, the mammalian proximal tubule is also the site of ammonia production and secretion of organic anions and cations. Expression of genes that encode transporters for ammonium (*rhbg*/*slc42a2*), organic ions (*slc22*), and organic anions (*slco*) provides compelling evidence in favor of the notion that similar activities are associated with the proximal tubules of the *Xenopus *pronephros. We conclude that the proximal tubule shares a strikingly high degree of structural and functional similarity with the mammalian proximal tubule.

### Evidence for an intermediate tubule compartment in the pronephros

Identification of a compartment that shares molecular characteristics with the intermediate tubule of the metanephros represents a major, unanticipated outcome of the present study. In birds and mammals, the intermediate tubule of the metanephric kidney gives rise to the thin limbs of Henle's loop, which are required to concentrate urine [2,3,46]. In contrast, the kidneys of larval and adult amphibians do not develop loops of Henle. Their urine is hypo-osmotic to the blood plasma, and they produce very dilute urine in freshwater [4]. Consistent with these findings, the existence of an intermediate tubule segment in the pronephric kidney had also previously been ruled out on the basis of ultrastructural studies [16]. Our arguments for postulating an intermediate tubule for the pronephros are based on molecular evidence and functional studies.

To date, only a few genes with specific expression in the thin limbs of Henle are known. These include aquaporin1 (*Aqp1*), the UT-A2 splice variant of the urea transporter *Slc14a2*, and claudin 8 (*Cldn8*) in the descending thin limb; and the kidney-specific chloride channel *Clcnka *in the ascending thin limb [47,48]. In *Xenopus*, the expression of *slc14a2 *could not be determined because no appropriate cDNA was available, and we failed to detect any pronephric expression of *aqp1 *(data not shown). In contrast, we were able to demonstrate *cldn8 *and *clcnk *expression in the intermediate tubules (Figure 5a,e). Furthermore, comparative gene expression analysis recently demonstrated that *Irx *homeobox transcription factors mark an intermediate compartment in the developing nephron of both the pronephros and the metanephros [22]. Interestingly, functional studies in *Xenopus *have shown that *irx3 *is required for intermediate tubule formation [22]. Taken together, molecular evidence and functional studies demonstrate the existence of a patterning mechanism for intermediate tubule formation at the level of the *Xenopus *pronephros. Despite sharing molecular similarities with the mammalian thin limb (*cldn8 *and *clckn *expression), the intermediate tubules of the *Xenopus *pronephric kidneys have also acquired characteristics of the distal tubule, which manifests as proximal expansion of the expression domains of the distal tubule marker genes *slc12a1*, *slc12a6*, and *kcnj1 *(Figure 7). It therefore appears that the intermediate tubules of the pronephros have evolved to function in the reabsorption of salts and ions.

### The *Xenopus *distal tubule shares similarities with the mammalian thick ascending limb and distal convoluted tubule

The mammalian distal tubule consists of two segments, TAL and DCT. The TAL remains impermeable to water and reabsorbs up to 25% of the filtered sodium and chloride via the Na-K-Cl transporter Slc12a1. Net movement of sodium across the TAL requires the recycling of potassium via the potassium channel Kcnj1 and the transport of chloride by the kidney-specific Clcnkb [49]. The TAL is also a major site of renal magnesium reabsorption, which occurs predominantly through a paracellular pathway and requires claudin 16/paracellin-1 (Cldn16) function [50]. Remarkably, we found that the DT1 segment of the *Xenopus *pronephros expresses the same set of genes mentioned above, which suggests that it is largely analogous to the TAL.

The transition from TAL to DCT is characterized by the sharp change in gene expression from *Slc12a1 *to the thiazide-sensitive Na-Cl transporter *Slc12a3 *(*NCC*) in different species of mammals [51]. Interestingly, this highly characteristic feature can also be observed in the *Xenopus *pronephros, where the same transition defines the border between DT1 and DT2. In addition to its role in sodium chloride reabsorption, the DCT also regulates the pH by absorbing bicarbonate and it secretes protons into the urine. The expression of the bicarbonate transporter *slc4a2 *in the DT2 segment suggests that similar functions are carried out by the *Xenopus *pronephros. Furthermore, the highly restricted expression of *rhcg*/*slc42a3 *indicates that the DT2 is capable of ammonium transport similar to the DCT [52,53]. Taken together, the present molecular evidence is in line with our proposal that the DT2 is the pronephric equivalent of the DCT in the metanephros.

### The pronephros harbors a simplified collecting duct system

In the mammalian metanephros, the collecting duct system is composed of the connecting tubule followed by the cortical and medullary collecting ducts. Our gene expression analysis of the pronephric kidney suggests that a single nephron segment links the distal tubule to the rectal diverticulum and the cloaca. The expression of the Na-Ca exchanger *slc8a1 *and the calcium-binding protein *calb1 *indicates that this segment shares molecular characteristics with the mammalian connecting tubule. We therefore refer to this segment of the pronephric nephron as the connecting tubule.

Despite the unexpected high degree of similarity in nephron organization, the pronephric and metanephric kidneys differ markedly in the organization of the collecting duct. We assessed the expression of several established marker genes of the mammalian collecting duct, such as *slc4a1*, *slc26a4*, and the aquaporins *aqp2*, *aqp3*, and *aqp4*, but we failed to detect any expression in the stage 35/36 pronephric kidney. At present, we cannot rule out the possibility that expression of these marker genes occurs only in older, more mature pronephric kidneys. In fact, when we assessed stage 40 embryos, we detected expression of the type A intercalated cell marker *slc4a1 *in the connecting tubule, the rectal diverticulum, and the cloaca; and the type B marker *slc26a4 *in the cloaca (DR and AWB, unpublished data). In contrast, no pronephric expression of *aqp2*, *aqp3*, and *aqp4 *was found. Although further analysis is still needed, these preliminary findings suggest that the maturation of intercalated cells may take place late after the onset of pronephric functions at stage 37/38.

The collecting duct system plays a major role in the final concentration of urine [7] and is therefore the last structure of the nephron that can modify the electrolyte and fluid balance in the mammalian body. The constraints on the body's physiology are different for *Xenopus *tadpoles living in fresh water. They are required to conserve salts and must excrete copious amounts of diluted urine to maintain water balance [4]. It is therefore not surprising that we failed to detect any evidence for pronephric expression of aquaporins. We conclude that the collecting duct system of pronephric kidney consists of a single nephron segment sharing similarities with the connecting tubule.

### The *Xenopus *pronephros as a novel model for human renal diseases

In recent years, mutations in various *SLC *genes and genes encoding ion channels and claudins have been discovered as the underlying causes of various forms of familial renal diseases in humans. The mutations impair important physiological functions that are confined to specific nephron segments, such as the proximal tubule, the TAL, and the DCT [54]. Interestingly, our large-scale gene expression analysis has now provided clear evidence that the *Xenopus *orthologs of at least 11 human renal disease genes were expressed in the equivalent nephron segments of the pronephric kidney (Table 3 and Additional data file 2). We therefore believe that the *Xenopus *embryo may become a useful model for rapid analysis of the basic molecular and regulatory processes that are involved in inherited human renal disease.

**Table 3:** Human renal disease genes that have *Xenopus *orthologs expressed in the pronephric kidney

Gene/function	Human renal disease	OMIM	Nephron segment	References
*SLC3A1*/Cystine, dibasic, and neutral amino acid transporter	Cystinuria	220100	PT	[90]
*SLC4A4*/Na-bicarbonate cotransporter	Proximal renal tubular acidosis with ocular abnormalities and mental retardation	604278	PT	[91]
*SLC5A2*/Na-glucose cotransporter	Renal glucosuria	233100	PT	[92]
*SLC6A19*/Neurotransmitter transporter	Hartnup disorder	234500	PT	[75,93]
*SLC7A7*/Cationic amino acid, y+ system	Lysinuric protein intolerance	222700	PT	[94,95]
*SLC34A3*/Na-phosphate cotransporter	Hereditary hypophosphatemic rickets with hypercalciuria	241530	PT	[96,97]
*CLDN16*/tight junction protein	Primary hypomagnesemia; childhood self-limiting hypercalciuria	248250	TAL, DCT	[49]
*SLC12A1*/Na-K-Cl transporter	Antenatal Bartter syndrome type 1	601678	TAL	[98]
*KCNJ1*/inwardly rectifying K channel	Antenatal Bartter syndrome type 2	241200	TAL, DCT, CNT, CD	[99]
*CLCNKB*/kidney Cl channel	Bartter syndrome type 3	607364	TAL, DCT, CNT, CD	[100]
*SLC12A3*/Na-Cl transporter	Gitelman syndrome	263800	DCT	[101]

CD, collecting duct; CNT, connecting tubule; DCT, distal convoluted tubule; OMIM, Online Mendelian Inheritance in Man; PT, proximal tubule; TAL, thick ascending limb.

## Conclusion

The present study revealed that the pronephric nephron is composed of four basic domains: proximal tubule, intermediate tubule, distal tubule, and connecting tubule. These domains share at the molecular level gene signatures that are typical of the mammalian nephron, and can be further subdivided into eight functionally distinct segments. The striking structural and functional similarities between the pronephric and the metanephric nephron revealed in this study will allow us to analyze in greater detail genes involved in nephron patterning, a process that remains poorly understood. Moreover, gene mutations underlying human renal disease can now be analyzed in a simple and cost-effective animal model.

## Materials and methods

### Gene nomenclature

The standard gene nomenclature suggested by Xenbase [55] and adopted by the National Center for Biotechnology Information for *X. laevis *genes is utilized rather than the original gene names to maximize compatibility with data available from other model systems. Where possible, *Xenopus *gene names are the same as their human orthologs.

### Identification and sequencing of *Xenopus *cDNAs

Screening of nonredundant and expressed sequence tag nucleotide databases for *X. laevis *cDNAs encoding *slc*, claudin, and aquaporin genes was performed at the National Center for Biotechnology Information BLAST website [56]. Initially, representative human *SLC *amino acid sequences were obtained from the Human Gene Organization database (HUGO) [57]. Later, reference sequences of human *slc*, claudin, and aquaporin genes were obtained from GenBank. The selected human sequences were used as protein queries in TBLASTN searches, which compare a protein sequence with the six-frame translations of a nucleotide database. Where more than one *Xenopus *cDNA sequence was retrieved, the cDNA encoding the longest open reading frame was selected for further analysis. Phylogeny and conservation of gene synteny were used as criteria for establishing the orthology of the selected *Xenopus *genes with the human counterparts. A full account of the database screens, synteny comparisons, and phylogenetic analyses will be published elsewhere (DR and AWB, unpublished data).

The *X. laevis *cDNAs were obtained from the German Resource Center for Genome Research RZPD/ImaGenes or the National Institute for Basic Biology (NIBB) in Japan. The cDNAs were obtained either as sequence-verified clones directly from RZPD/ImaGenes or sequenced in-house using the BigDye Terminator v3.1 Cycle Sequencing Kit (Applied Biosystems) and the 3130 Genetic Analyzer (Applied Biosystems, Foster City, CA, USA) DNA sequencer.

### *Xenopus *embryo manipulations and *in situ *hybridization

*In vitro *fertilization, culture, and staging of *Xenopus *embryos were performed as described [58,59,28]. Whole-mount *in situ *hybridizations and bleaching of stained *Xenopus *embryos were carried out using established protocols [59-61]. All pre-hybridization and post-hybridization washes were performed in nylon mesh baskets using a BioLane HTI *in situ *hybridization machine (Holle & Hüttner AG, Tübingen, Germany). For probe hybridization, the baskets with embryos were transferred to 15 ml Falcon tubes and hybridizations were performed in a water bath. Templates for cRNA probe synthesis were either plasmids linearized by restriction enzyme digestion or PCR products of the cDNA inserts generated by amplification with the appropriate T3, T7, or SP6 primers. Inserts cloned into the pDNRLib vector were amplified by PCR using the following primers: pDNRlib lower: 5'-GTC TAG AAA GCT TCT CGA GGG-3'; and pDNRlib upper: 5'-GGA CAT ATG CCC GGG AAT TCG GCC-3'. The resulting PCR products were subcloned into the pGEM-T Easy vector (Promega, Madison, WI, USA), in accordance with the pGEM-T Easy Vector System I protocol. Digoxigenin-labeled cRNA probes were either transcribed from linearized plasmids or made directly from PCR products using T7, T3, or SP6 polymerases (Roche, Basel, Switzerland). Sense strand controls were prepared from all plasmids and tested by *in situ *hybridization. The GenBank accession numbers of the cDNAs used for *in situ *hybridization are given in Additional data file 2. The GenBank accession numbers for the *Xenopus *aquaporin cDNAs are as follows: *aqp1 *(CD302300), *aqp2 *(AY151156), *aqp3 *(CA9711164), and *aqp4 *(BG515560). For each reported gene, at least 40 embryos were examined.

### Contour model of the pronephric nephron and marker gene mapping

A first schematic representation of the contour of the stage 35/36 nephron was developed from *Xenopus *embryos stained by standard single-color whole-mount *in situ *hybridization with a combination of digoxigenin-labeled probes for the pronephric marker genes *fxyd2 *[45], *pax2 *[62], and *wnt4 *[61]. Two dozen stained embryos were inspected to generate a two-dimensional contour drawing of the nephron onto paper. Refinements to the initial contour model were made after inspection of hundreds of embryos stained with other pronephric marker genes. The final contour model of the nephron shown in Figure 2a was made with Illustrator CS2 (Adobe, San Jose, CA, USA).

The pronephric expression patterns of the marker genes were projected onto the contour model to define the segments of the nephron. Unambiguous morphological features, such as the nephrostomes, a characteristically broad proximal tubule domain known as common tubule [29] (subsequently named PT3) and the looped part of the pronephric nephron (IT1, IT2, and DT1) were used as landmarks to identify the relative location of the boundaries of the expression domains. The final borders between the nephron segments are defined by the boundaries of multiple marker genes.

### Murine tissue preparation

Kidneys of male adult C57BL/6J mice were collected in ice-cold phosphate-buffered saline and subsequently transferred into 4% paraformaldehyde for fixation at 4°C for at least 3 days. The kidneys were dehydrated in an ethanol series and passed through xylene into paraffin with each step lasting a full day. Paraffinized kidneys were sectioned in transversal orientation at 8 nm thickness using a Leica RM 2165 microtome (Leica, Wetzlar, Germany). Sections were deparaffinized in X-tra-Solve (Medite Histotechnik, Burgdorf, Germany), rehydrated, and then treated according to the protocol for fixation and acetylation of fresh frozen sections as reported by Yaylaoglu and coworkers [63].

### Murine *in situ *hybridization

Mouse cDNAs for *Slc5a1 *and *Cldn8 *were kindly provided by Alexandre Reymond (University of Geneva, Geneva, Switzerland). *Slc7a13 *and *Kcnj1 *cDNAs were obtained from RZPD/ImaGenes. Clone-derived template sequences were amplified by PCR using standard primers for T7 and SP6. PCR products were sequence verified and directly used for *in vitro *transcription. Templates for all other genes were generated by PCR from a cDNA pool representing a variety of embryonic and postembryonic tissues, as described previously [63]. The primers consisted of 25 nucleotides of gene-specific sequence linked to SP6, T3, or T7 polymerase promoter sites. Specific primer sequences for the individual genes can be obtained from the GenePaint database [64].

Riboprobe synthesis and robotic *in situ *hybridization were carried out using established protocols [63]. The *in situ *hybridization protocol includes a tyramine-biotin amplification reaction step. The protocol was adjusted for adult kidney paraffin sections by increasing the proteinase K concentration to 10 mg/ml, using a probe concentration of 300 ng/ml, and increasing the time of color reaction to three times 12 minutes.

### Photography and computer graphics

For *Xenopus*, photographs of stained embryos were taken digitally with an AxioCam Colour camera mounted on a Zeiss SteREO Lumar V12 stereoscopic microscope using the Axio Vision 4.5 (Zeiss, Feldbach, Switzerland) software. Image processing was carried out using Adobe Photoshop CS2 and Adobe InDesign CS2 software. Schematic figures were drawn using Adobe Illustrator CS2.

Stained slides of mouse kidney sections were scanned using a Leica DM-RXA2 microscope equipped with the Leica electronic focusing system and a motorized stage (Märzhäuser, Wetzlar-Steindorf, Germany). Brightfield images were collected with a CCD camera (Hitachi, Tokyo, Japan) and a 10× objective (NA 0.40; Leica). Custom-made software was used to drive stage and camera [65]. The kidney sections were too large to be photographed as a whole. Therefore, multiple images were taken. Each image was stored as a bitmap file and individual images were assembled into a mosaic image that was cropped, properly oriented, and saved as a TIFF file. The resulting TIFF images with a resolution of 1.6 μm/pixel were deposited in the GenePaint database along with metadata such as specimen, gene name, and probe sequence.

### Informatics resources

The programming underlying the XGEbase web resource follows a classical three-tier architecture. The first tier is a web-based user interface providing an access point for users to browse gene expression patterns in the developing pronephros. The second tier is the application layer, which is responsible for process management tasks. It is implemented using Java and Java Server Faces (JSF) technologies. It receives parameters from the client's machine to query the core database and return dynamically generated web pages based on the values retrieved from the database. The third tier is the database management system (DBMS) and core database. The DBMS is responsible for creation, maintenance, and interrogation of data stored in the database. The application layer communicates with this layer in order to retrieve specific data. A MySQL relational DBMS (version 5.0.4) [66] has been used to implement this layer.

In XGEbase, the pronephric expression patterns are fully annotated in accordance with the nephron segmentation model (Figure 2a), and the strength of the *in situ *hybridization signal is given for each anatomical structure. Important meta-data, including GenBank accession numbers, *in situ *hybridization probe details, and specimen preparation, are provided for each gene. Furthermore, links are provided to other related database such as Xenbase, Online Mendelian Inheritance in Man (OMIM), Entrez, GenCards, Gene Ontology (GO), HUGO Gene Nomenclature Committee (HGNC), and GenitoUrinary Development Molecular Anatomy Project (GUDMAP). The database interface allows for browsing per gene (individual level) or per segment of the pronephric kidney (comparative level). It is also possible to search images by querying both the segment and the signal intensity. The database will be expanded to include other gene families with a focus on terminal differentiation markers.

## Abbreviations

DBMS, database management system; DCT, distal convoluted tubule; DT, distal tubule; EuReGene, European Renal Genome Project; hpf, hours postfertilization; IT, intermediate tubule; PCR, polymerase chain reaction; PT, proximal tubule; TAL, thick ascending limb; XGEbase, EuReGene *Xenopus *Gene Expression Database.

## Authors' contributions

AWB conceived of the project. DR performed bioinformatics analysis, and designed and carried out the *Xenopus slc in situ *hybridization project. FB and DR carried out the claudin *in situ *hybridization project. AES and DR performed the aquaporin *in situ *hybridization project. DR and LR annotated and analyzed the *Xenopus *gene expression patterns. DR, LR, BK, and AWB developed the pronephric nephron organization model. LG and QJ performed the mouse *in situ *hybridizations. DR and BK annotated the mouse kidney gene expression patterns. DC, CT, DR, AWB, and DRD developed the *Xenopus *gene expression database (XGEbase). DR and AWB wrote the paper. All authors read and approved the final manuscript.

## Additional data files

The following additional data are available with the online version of this paper. Additional data file 1 is a table listing marker gene expression in the developing *Xenopus *pronephric kidney at stages 25, 29/30, 35/36, and 40. Additional data file 2 is a table containing the annotation of marker gene expression in the *Xenopus *stage 35/36 pronephric kidney. Additional data file 3 is a table listing marker genes expressed in the proximal tubule of the stage 35/36 pronephric kidney. Additional data file 4 is table listing marker genes expressed in the intermediate tubule of the stage 35/36 pronephric kidney. Additional data file 5 is a table listing marker genes expressed in the distal tubule of the stage 35/36 pronephric kidney. Additional data file 6 is a table listing marker genes that are expressed in the connecting tubule of the stage 35/36 pronephric kidney.

## Supplementary Material

Additional data file 1 Presented is a table listing marker gene expression in the developing *Xenopus *pronephric kidney at stages 25, 29/30, 35/36, and 40, as determined by whole-mount *in situ *hybridization. None of the genes analyzed were expressed in the stage 20 pronephric anlage. The expression levels were graded as follows: possible (+/-), present (+), and strong (++). The GenBank accession numbers of the cDNAs used for *in situ *hybridization probe synthesis are provided. Click here for file

Additional data file 2 Presented is a table containing the annotation of marker gene expression in the *Xenopus *stage 35/36 pronephric kidney. The expression levels in the pronephros are indicated as follows: absent (-), possible (+/-), present (+), and strong (++). Abbreviations: CT, connecting tubule; DT, distal tubule; IT, intermediate tubule; PT, proximal tubule. Click here for file

Additional data file 3 Presented is a table listing marker genes expressed in the proximal tubule of the stage 35/36 pronephric kidney, as determined by whole-mount *in situ *hybridization. Genes expressed exclusively in this compartment are indicated with asterisks. Click here for file

Additional data file 4 Presented is a table listing marker genes expressed in the intermediate tubule of the stage 35/36 pronephric kidney, as determined by whole-mount *in situ *hybridization. Genes expressed exclusively in this compartment are indicated with asterisks. Click here for file

Additional data file 5 Presented is a table listing marker genes expressed in the distal tubule of the stage 35/36 pronephric kidney, as determined by whole-mount *in situ *hybridization. Genes expressed exclusively in this compartment are indicated with asterisks. Click here for file

Additional data file 6 Presented is a table listing marker genes expressed in the connecting tubule of the stage 35/36 pronephric kidney, as determined by whole-mount *in situ *hybridization. Genes expressed exclusively in this compartment are indicated with asterisks. Click here for file
