# Supplementary material for: Organization of the pronephric kidney revealed by large-scale gene expression mapping
Source: Genome Biol. 2008 May 20;9(5):R84. doi: 10.1186/gb-2008-9-5-r84 (PMC2441470; doi:10.1186/gb-2008-9-5-r84)
Supplement: Additional data file 1 — Presented is a table listing marker gene expression in the developing Xenopus pronephric kidney at stages 25, 29/30, 35/36, and 40, as determined by whole-mount in situ hybridization. None of the genes analyzed were expressed in the stage 20 pronephric anlage. The expression levels were graded as follows: possible (+/-), present (+), and strong (++). The GenBank accession numbers of the cDNAs used for in situ hybridization probe synthesis are provided. [file gb-2008-9-5-r84-S1.pdf]

Addition data file 1: Marker gene expression at selected stages of pronephric development

Genes expressed in the stage 25 pronephric kidney

Gene	Synonyms	GenBank acc. no.	Expression
slc2a2	GLUT2	BC070704.1	+
slc5a1	SGLT1, D22S675	CA974591.1	+/-
slc6a14	-	BU911733.1	++
slc7a3	CAT-3, ATRC3, FLJ14541	BC042222.1	+
slc7a7	y+LAT-1	BC072040.1	+
slc12a1	NKCC2	CF520237.1	++
slc19a2	TRMA, THTR1	BC070848.1	+
slco2a1	SLC21A2, PGT, OATP2A1	BC060473.1	+/-
slc30a8	-	BG037315.1	+
slc38a7	-	BC076791.1	+/-
cldn6	-	BC077402.1	++

Addition data file 1: Marker gene expression at selected stages of pronephric development

Genes expressed in the stage 29/30 pronephric kidney

Gene	Synonyms	GenBank acc. no.	Expression
slc2a2	GLUT2	BC070704.1	++
slc2a4	GLUT4	BC073012.1	+
slc2a8	GLUTX1, GLUT8	CB593014.1	+
slc2a10	GLUT10	BC073721.1	+
slc2a13	HMIT	BC092027.1	+
slc3a2	4T2HC, 4F2, NACAE	BC042234.1	+/-
slc4a2	EPB3L1, AE2, HKB3	BG348033.1	+/-
slc4a4	NBC1, HNBC1, NBC2, pNBC, hhNMC, SLC4A5	BU905206.1	++
slc4a7	SLC4A6, NBC3, SBC2	BC070701.1	++
slc4a11	CHED2, dJ794I6.2, BTR1	BU904542.1	++
slc5a1	SGLT1, D22S675	CA974591.1	++
slc5a2	SGLT2	BC081106.1	++
slc5a5	NIS	BC077614.1	+/-
slc5a6	SMVT	BF611525.1	++
slc5a8	AIT	BC060005.1	++
slc5a9	SGLT4	CA788193.1	++
slc5a11	KST1, SMIT2, SGLT6	AB008225.1	++
slc6a9	-	BQ737355.1	+
slc6a14	-	BU911733.1	++
slc6a19	-	BC081075.1	++
slc7a3	CAT-3, ATRC3, FLJ14541	BC042222.1	+
slc7a7	y+LAT-1	BC072040.1	++
slc7a8	LPI-PC1, LAT2	BC044971.1	+
slc8a1	NCX1	BG371210.1	+
slc9a6	NHE6, KIAA0267	CA987997.1	+
slc12a1	NKCC2	CF520237.1	++
slc12a6	KCC3, ACCPN	BC054325.1	+
slc13a5	NACT	BC077435.1	+
slc15a2	PEPT2	BQ386718.1	+
slc15a4	PHT1, PTR4	BC079971.1	+
slc16a1	MCT, MCT1	BC070980.1	+
slc16a12	MCT12	BC074222.1	++
slc17a5	SIASD, AST, SD, ISSD, NSD, SIALIN, SLD	BI445533.1	+
slc19a1	FOLT	BC073675.1	+
slc19a2	TRMA, THTR1	BC070848.1	++
slc20a1	GLVR1, PiT-1, Glvr-1	BU903168.1	++
slco2a1	SLC21A2, PGT, OATP2A1	BC060473.1	+/-
slc22a5	CDSP, OCTN2, SCD	BC056014.1	++
slc25a1	SLC20A3,CTP	BC041303.1	+
slc25a5	ANT2, T2, 2F1, T3	BC043821.1	+
slc25a11	SLC20A4,OGC	BC072308.1	+
slc25a22	GC1, FLJ13044	BC063272.1	+
slc25a32	MFTC	BC087370.1	+
slc25a39	FLJ22407, CGI-69	BC073249.1	+
slc25a44	FLJ90431, KIAA0446	BC076803.1	+
slc26a1	SAT-1, EDM4	BU904894.1	+
slc26a6	-	BC075145.1	++
slc27a7	-	BX850807.1	+
slc29a3	ENT3, FLJ11160	BC077451.1	+
slc30a7	ZnTL2, ZNT7	BC070769.1	++
slc30a8	-	BG037315.1	++

Addition data file 1: Marker gene expression at selected stages of pronephric development

Gene	Synonyms	GenBank acc. no.	Expression
slc30a9	C4orf1, HUEL, ZNT9, GAC63	BC078104.1	++
slc31a1	COPT1, hCTR1, CTR1	BC075178.1	++
slc31a2	COPT2, hCTR2, CTR2	CA971177.1	+
slc33a1	ACATN, AT-1	BC068928.1	++
slc34a3	NPTIIc, FLJ38680	BC082530.1	++
slc35a1	CMPST, hCST	CA791665.1	+
slc35a5	FLJ20730	BC078070.1	++
slc35f2	FLJ13018	BC084761.1	+
slc36a1	LYAAT-1, PAT1, TRAMD3	BC070857.1	+
slc37a2	FLJ00171	BC042235.1	++
slc38a7	-	BC076791.1	+
slc39a6	LIV-1	CA788000.1	++
slc39a8	BIGM103	BP686084.2	+
rhcg	SLC42A3, C15orf6, RHGK, PDRC2	BC078079.1	+
cldn3	C7orf1, CPETR2,RVP1	BC079722.1	++
cldn6	-	BC077402.1	++
cldn12	-	BC088962.1	+
cldn14	DFNB29	BC074122.1	+
cldn19	-	BC082674.1	++
clcnk	Clcnka, Clcnkb	NM_001085839	++
kcnj1	Kir1.1, ROMK1	CF522101.1	+

Addition data file 1: Marker gene expression at selected stages of pronephric development

Genes expressed in the stage 35/36 pronephric kidney

Gene	Synonyms	GenBank acc. no.	Expression
slc1a1	EAAC1, EAAT3	CV079713.1	++
slc2a2	GLUT2	BC070704.1	++
slc2a4	GLUT4	BC073012.1	+
slc2a8	GLUTX1, GLUT8	CB593014.1	+
slc2a10	GLUT10	BC073721.1	+
slc2a11	GLUT11	CF519465.1	++
slc2a13	HMIT	BC092027.1	+
slc3a1	CSNU1, D2H, RBAT	BU903456.1	++
slc3a2	4T2HC, 4F2, NACAE	BC042234.1	++
slc4a2	EPB3L1, AE2, HKB3	BG348033.1	+
slc4a4	NBC1, HNBC1, NBC2, pNBC, hhNMC, SLC4A5	BU905206.1	++
slc4a7	SLC4A6, NBC3, SBC2	BC070701.1	++
slc4a11	CHED2, dJ794I6.2, BTR1	BU904542.1	++
slc5a1	SGLT1, D22S675	CA974591.1	++
slc5a2	SGLT2	BC081106.1	++
slc5a5	NIS	BC077614.1	+/-
slc5a6	SMVT	BF611525.1	++
slc5a8	AIT	BC060005.1	++
slc5a9	SGLT4	CA788193.1	++
slc5a11	KST1, SMIT2, SGLT6	AB008225.1	++
slc6a9	-	BQ737355.1	+
slc6a13	GAT2	BC060418.1	++
slc6a14	-	BU911733.1	++
slc6a19	-	BC081075.1	++
slc7a6	y+LAT-2, KIAA0245, LAT3, LAT-2	BQ736312.1	+
slc7a7	y+LAT-1	BC072040.1	++
slc7a8	LPI-PC1, LAT2	BC044971.1	++
slc7a13	AGT-1, XAT2	BC060020.1	++
slc8a1	NCX1	BG371210.1	+
slc9a1	APNH, NHE1	BU906256.1	+/-
slc9a6	NHE6, KIAA0267	CA987997.1	++
slc12a1	NKCC2	CF520237.1	++
slc12a3	-	CA790325.1	+
slc12a6	KCC3, ACCPN	BC054325.1	++
slc13a3	NADC3, SDCT2	BC075138.1	+
slc13a5	NACT	BC077435.1	++
slc15a2	PEPT2	BQ386718.1	++
slc15a4	PHT1, PTR4	BC079971.1	++
slc16a1	MCT, MCT1	BC070980.1	++
slc16a6	MCT6, MCT7	BC047967.1	++
slc16a7	MCT2	BJ059209.1	+
slc16a9	FLJ43803, MCT9	CF520266.1	+
slc16a12	MCT12	BC074222.1	++
slc17a5	SIASD, AST, SD, ISSD, NSD, SIALIN, SLD	BI445533.1	+
slc19a1	FOLT	BC073675.1	++
slc19a2	TRMA, THTR1	BC070848.1	++
slc20a1	GLVR1, PiT-1, Glvr-1	BU903168.1	++
slco2a1	SLC21A2, PGT, OATP2A1	BC060473.1	++
slc22a5	CDSP, OCTN2, SCD	BC056014.1	++
slc22a6	ROAT1, PAHT, OAT1	BC081057.1	++

Addition data file 1: Marker gene expression at selected stages of pronephric development

Gene	Synonyms	GenBank acc. no.	Expression
slc22a13	ORCTL3, OCTL1, OCTL3	CB559054.1	++
slc23a2	SLC23A1, SVCT2, KIAA0238, YSPL2	CF522441.1	+
slc25a1	SLC20A3,CTP	BC041303.1	++
slc25a3	PHC	BC046849.1	+
slc25a4	PEO3, PEO2, ANT1, T1	BC072091.1	+
slc25a5	ANT2, T2, 2F1, T3	BC043821.1	+
slc25a10	DIC	BC070665.1	++
slc25a11	SLC20A4,OGC	BC072308.1	+
slc25a20	CACT, CAC	BC043827.1	++
slc25a22	GC1, FLJ13044	BC063272.1	+
slc25a32	MFTC	BC087370.1	+
slc25a39	FLJ22407, CGI-69	BC073249.1	+/-
slc25a44	FLJ90431, KIAA0446	BC076803.1	+
slc26a1	SAT-1, EDM4	BU904894.1	++
slc26a6	-	BC075145.1	++
slc26a11	-	CA988173.1	++
slc27a7	-	BX850807.1	+
slc28a1	CNT1	CD098603.1	++
slc29a3	ENT3, FLJ11160	BC077451.1	++
slc30a7	ZnTL2, ZNT7	BC070769.1	++
slc30a8	-	BG037315.1	++
slc30a9	C4orf1, HUEL, ZNT9, GAC63	BC078104.1	++
slc31a1	COPT1, hCTR1, CTR1	BC075178.1	++
slc31a2	COPT2, hCTR2, CTR2	CA971177.1	++
slc33a1	ACATN, AT-1	BC068928.1	++
slc34a3	NPTIIc, FLJ38680	BC082530.1	++
slc35a1	CMPST, hCST	CA791665.1	++
slc35a4	-	EB646007.1	+
slc35a5	FLJ20730	BC078070.1	++
slc35b2	UGTrel4	BC044702.1	+
slc35c1	FUCT1, FLJ11320	BJ039584.1	+
slc35f2	FLJ13018	BC084761.1	++
slc36a1	LYAAT-1, PAT1, TRAMD3	BC070857.1	++
slc37a2	FLJ00171	BC042235.1	++
slc38a2	SAT2, ATA2, KIAA1382, SNAT2	BC077990.1	+
slc38a7	-	BC076791.1	++
slc39a6	LIV-1	CA788000.1	+/-
slc39a8	BIGM103	BP686084.2	+
rhbg	SLC42A2	BC078079.1	+
rhcg	SLC42A3, C15orf6, RHGK, PDRC2	BC084943.1	++
slc43a2	MGC34680	BC074223.1	++
cldn3	C7orf1, CPETR2,RVP1	BC079722.1	++
cldn4	CPETR, CPETR1, CPE-R, WBSCR8, hCPE-R	BC099009.1	++
cldn6	-	BC077402.1	++
cldn8	-	DR877133.1	+
cldn12	-	BC088962.1	+
cldn14	DFNB29	BC074122.1	++
cldn16	PCLN1	CD100665.1	++
cldn19	-	BC082674.1	++
clcnk	Clcnka, Clcnkb	NM_001085839	++
kcnj1	Kir1.1, ROMK1	CF522101.1	++
calb1	-	U76636.1	+

Addition data file 1: Marker gene expression at selected stages of pronephric development

Genes expressed in the stage 40 pronephric kidney

Gene	Synonyms	GenBank acc. no.	Expression
slc1a1	EAAC1, EAAT3	CV079713.1	++
slc2a2	GLUT2	BC070704.1	++
slc2a3	GLUT3	BC049174.1	+
slc2a4	GLUT4	BC073012.1	+
slc2a8	GLUTX1, GLUT8	CB593014.1	+/-
slc2a11	GLUT11	CF519465.1	++
slc2a13	HMIT	BC092027.1	+
slc3a1	CSNU1, D2H, RBAT	BU903456.1	++
slc3a2	4T2HC, 4F2, NACAE	BC042234.1	+/-
slc4a1	EPB3, AE1, DI, WD, RTA1A, CD233, FR, SW, WR	BC072920.1	++
slc4a2	EPB3L1, AE2, HKB3	BG348033.1	+
slc4a4	NBC1, HNBC1, NBC2, pNBC, hhNMC, SLC4A5	BU905206.1	++
slc4a7	SLC4A6, NBC3, SBC2	BC070701.1	++
slc4a11	CHED2, dJ794I6.2, BTR1	BU904542.1	++
slc5a1	SGLT1, D22S675	CA974591.1	++
slc5a2	SGLT2	BC081106.1	++
slc5a6	SMVT	BF611525.1	++
slc5a8	AIT	BC060005.1	++
slc5a9	SGLT4	CA788193.1	++
slc5a11	KST1, SMIT2, SGLT6	AB008225.1	++
slc6a9	-	BQ737355.1	+
slc6a13	GAT2	BC060418.1	++
slc6a14	-	BU911733.1	+
slc6a19	-	BC081075.1	++
slc7a5	LAT1, E16, D16S469E, MPE16, CD98	BC060751.1	+/-
slc7a6	y+LAT-2, KIAA0245, LAT3, LAT-2	BQ736312.1	+
slc7a7	y+LAT-1	BC072040.1	++
slc7a8	LPI-PC1, LAT2	BC044971.1	+
slc7a13	AGT-1, XAT2	BC060020.1	++
slc8a1	NCX1	BG371210.1	++
slc9a1	APNH, NHE1	BU906256.1	+
slc9a6	NHE6, KIAA0267	CA987997.1	++
slc12a1	NKCC2	CF520237.1	++
slc12a3	-	CA790325.1	++
slc12a6	KCC3, ACCPN	BC054325.1	+
slc13a3	NADC3, SDCT2	BC075138.1	++
slc13a5	NACT	BC077435.1	++
slc15a2	PEPT2	BQ386718.1	++
slc15a4	PHT1, PTR4	BC079971.1	+
slc16a1	MCT, MCT1	BC070980.1	++
slc16a3	MCT3, MCT4	BC074173.1	+/-
slc16a6	MCT6, MCT7	BC047967.1	++
slc16a7	MCT2	BJ059209.1	++
slc16a9	FLJ43803, MCT9	CF520266.1	++
slc16a12	MCT12	BC074222.1	++
slc17a5	SIASD, AST, SD, ISSD, NSD, SIALIN, SLD	BI445533.1	+
slc19a1	FOLT	BC073675.1	+
slc19a2	TRMA, THTR1	BC070848.1	++

Addition data file 1: Marker gene expression at selected stages of pronephric development

Gene	Synonyms	GenBank acc. no.	Expression
slc20a1	GLVR1, PiT-1, Glvr-1	BU903168.1	+
slc22a2	02.Okt	BC061664.1	++
slc22a5	CDSP, OCTN2, SCD	BC056014.1	++
slc22a6	ROAT1, PAHT, OAT1	BC081057.1	++
slc22a13	ORCTL3, OCTL1, OCTL3	CB559054.1	++
slc22a15	FLIPT1	BC068683.1	+
slc23a2	SLC23A1, SVCT2, KIAA0238, YSPL2	CF522441.1	++
slc25a1	SLC20A3,CTP	BC041303.1	++
slc25a3	PHC	BC046849.1	++
slc25a10	DIC	BC070665.1	++
slc25a11	SLC20A4,OGC	BC072308.1	+
slc25a20	CACT, CAC	BC043827.1	+
slc25a32	MFTC	BC087370.1	+/-
slc25a39	FLJ22407, CGI-69	BC073249.1	+
slc25ucp2	SLC25A8	BC086297.1	++
slc26a1	SAT-1, EDM4	BU904894.1	++
slc26a6	-	BC075145.1	++
slc26a11	-	CA988173.1	+
slc28a1	CNT1	CD098603.1	++
slc29a2	ENT2, HNP36, DER12	BC073653.1	++
slc30a7	ZnTL2, ZNT7	BC070769.1	+
slc30a8	-	BG037315.1	+
slc30a9	C4orf1, HUEL, ZNT9, GAC63	BC078104.1	+
slc31a1	COPT1, hCTR1, CTR1	BC075178.1	++
slc31a2	COPT2, hCTR2, CTR2	CA971177.1	++
slc33a1	ACATN, AT-1	BC068928.1	+
slc34a3	NPTIIc, FLJ38680	BC082530.1	++
slc35a1	CMPST, hCST	CA791665.1	++
slc35a5	FLJ20730	BC078070.1	++
slc35b2	UGTrel4	BC044702.1	+
slc35f2	FLJ13018	BC084761.1	++
slc36a1	LYAAT-1, PAT1, TRAMD3	BC070857.1	+
slc37a2	FLJ00171	BC042235.1	++
slc38a2	SAT2, ATA2, KIAA1382, SNAT2	BC077990.1	++
slc38a7	-	BC076791.1	+
slc39a6	LIV-1	CA788000.1	+/-
slc39a8	BIGM103	BP686084.2	++
rhbg	SLC42A2	BC078079.1	++
rhcg	SLC42A3, C15orf6, RHGK, PDRC2	BC084943.1	++
rhd	RH, Rh30a, Rh4, RhPI, RhII, DIIIc, CD240D	AY207444.1	++
slc43a2	MGC34680	BC074223.1	+
cldn3	C7orf1, CPETR2,RVP1	BC079722.1	++
cldn4	CPETR, CPETR1, CPE-R, WBSCR8, hCPE-R	BC099009.1	++
cldn6	-	BC077402.1	+
cldn8	-	DR877133.1	+
cldn12	-	BC088962.1	+
cldn14	DFNB29	BC074122.1	++
cldn16	PCLN1	CD100665.1	++
cldn19	-	BC082674.1	++
clcnk	Clcnka, Clcnkb	NM_001085839	++
kcnj1	Kir1.1, ROMK1	CF522101.1	++
calb1	-	U76636.1	+
