# Supplementary material for: Organization of the pronephric kidney revealed by large-scale gene expression mapping
Source: Genome Biol. 2008 May 20;9(5):R84. doi: 10.1186/gb-2008-9-5-r84 (PMC2441470; doi:10.1186/gb-2008-9-5-r84)
Supplement: Additional data file 2 — Presented is a table containing the annotation of marker gene expression in the Xenopus stage 35/36 pronephric kidney. The expression levels in the pronephros are indicated as follows: absent (-), possible (+/-), present (+), and strong (++). Abbreviations: CT, connecting tubule; DT, distal tubule; IT, intermediate tubule; PT, proximal tubule. [file gb-2008-9-5-r84-S2.pdf]

Additional data file 2: Summary of marker gene expression domains for the *Xenopus* stage 35/36 pronephros

Gene	Synonyms	GenBank acc. no.	PT1	PT2	PT3	IT1	IT2	DT1	DT2	CT
slc1a1	EAAC1, EAAT3	CV079713.1	-	-	++	-	-	-	-	-
slc2a2	GLUT2	BC070704.1	++	++	++	-	-	-	-	-
slc2a4	GLUT4	BC073012.1	-	-	-	+	+	+	+	+
slc2a8	GLUTX1, GLUT8	CB593014.1	+	+	+	+/-	+/-	-	-	-
slc2a10	GLUT10	BC073721.1	+	+	+	-	-	-	-	-
slc2a11	GLUT11	CF519465.1	-	++	++	-	-	-	-	-
slc2a13	HMIT	BC092027.1	+	+	+	-	-	-	-	-
slc3a1	CSNU1, D2H, RBAT	BU903456.1	++	++	++	-	-	-	-	-
slc3a2	4T2HC, 4F2, NACAE	BC042234.1	+	+	+/-	+/-	+/-	-	-	-
slc4a2	EPB3L1, AE2, HKB3	BG348033.1	+	+	+	-	-	+/-	+	+/-
slc4a4	NBC1, HNBC1, NBC2, pNBC, hhNMC, SLC4A5	BU905206.1	++	++	+	-	-	++	-	-
slc4a7	SLC4A6, NBC3, SBC2	BC070701.1	++	++	++	-	-	-	-	-
slc4a11	CHED2, dJ794I6.2, BTR1	BU904542.1	++	++	+/-	++	++	++	-	-
slc5a1	SGLT1, D22S675	CA974591.1	-	++	++	+	+	-	-	-
slc5a2	SGLT2	BC081106.1	++	++	-	-	-	-	-	-
slc5a5	NIS	BC077614.1	+/-	+/-	+/-	-	-	-	-	-
slc5a6	SMVT	BF611525.1	++	++	++	-	-	-	-	-
slc5a8	AIT	BC060005.1	-	++	++	-	++	++	-	-
slc5a9	SGLT4	CA788193.1	-	++	++	-	-	-	-	-
slc5a11	KST1, SMIT2, SGLT6	AB008225.1	++	++	++	-	-	-	-	-
slc6a9	-	BQ737355.1	-	-	-	+	-	-	-	-
slc6a13	GAT2	BC060418.1	++	++	++	-	-	-	-	-
slc6a14	-	BU911733.1	-	-	++	++	++	++	++	++

Additional data file 2: Summary of marker gene expression domains for the *Xenopus* stage 35/36 pronephros

Gene	Synonyms	GenBank acc. no.	PT1	PT2	PT3	IT1	IT2	DT1	DT2	CT
slc6a19	-	BC081075.1	++	++	-	-	-	-	-	-
slc7a6	y+LAT-2, KIAA0245, LAT3, LAT-2	BQ736312.1	+	+/-	+/-	+	+/-	+	+	+
slc7a7	y+LAT-1	BC072040.1	++	+/-	-	-	-	-	-	-
slc7a8	LPI-PC1, LAT2	BC044971.1	++	+/-	-	-	-	-	-	-
slc7a13	AGT-1, XAT2	BC060020.1	-	+/-	++	-	-	-	-	-
slc8a1	NCX1	BG371210.1	-	-	-	-	-	-	-	+
slc9a1	APNH, NHE1	BU906256.1	+/-	+/-	+/-	+/-	+/-	+/-	+/-	+/-
slc9a6	NHE6, KIAA0267	CA987997.1	-	+	+	-	-	-	-	-
slc12a1	NKCC2	CF520237.1	-	-	-	++	++	++	-	-
slc12a3	-	CA790325.1	-	-	-	-	-	-	++	++
slc12a6	KCC3, ACCPN	BC054325.1	+	+	+	+	+/-	+	+	-
slc13a3	NADC3, SDCT2	BC075138.1	-	+	+	-	-	-	-	-
slc13a5	NACT	BC077435.1	++	++	++	-	-	-	-	-
slc15a2	PEPT2	BQ386718.1	++	++	-	-	-	-	-	-
slc15a4	PHT1, PTR4	BC079971.1	-	++	++	-	-	-	-	-
slc16a1	MCT, MCT1	BC070980.1	+	+	-	-	-	-	-	-
slc16a6	MCT6, MCT7	BC047967.1	+	+	+	+	-	++	++	++
slc16a7	MCT2	BJ059209.1	-	-	-	++	++	++	++	++
slc16a9	FLJ43803, MCT9	CF520266.1	-	++	++	-	-	-	-	-
slc16a12	MCT12	BC074222.1	-	++	++	-	-	-	-	-
slc17a5	SIASD, AST, SD, ISSD, NSD, SIALIN, SLD	BI445533.1	+	+	+	-	-	-	-	-
slc19a1	FOLT	BC073675.1	++	++	++	-	-	-	-	-
slc19a2	TRMA, THTR1	BC070848.1	+/-	+/-	+/-	++	+	-	+	+/-
slc20a1	GLVR1, PiT-1, Glvr-1	BU903168.1	-	-	-	+	-	-	-	-
slco2a1	SLC21A2, PGT, OATP2A1	BC060473.1	-	++	++	+	+	+	-	-

Additional data file 2: Summary of marker gene expression domains for the *Xenopus* stage 35/36 pronephros

Gene	Synonyms	GenBank acc. no.	PT1	PT2	PT3	IT1	IT2	DT1	DT2	CT
slc22a5	CDSP, OCTN2, SCD	BC056014.1	++	++	++	-	-	-	-	-
slc22a6	ROAT1, PAHT, OAT1	BC081057.1	-	++	++	-	-	-	-	-
slc22a13	ORCTL3, OCTL1, OCTL3	CB559054.1	++	++	-	-	-	-	-	-
slc23a2	SLC23A1, SVCT2, KIAA0238, YSPL2	CF522441.1	++	++	+/-	-	-	-	-	-
slc25a1	SLC20A3,CTP	BC041303.1	++	++	++	-	-	-	-	-
slc25a3	PHC	BC046849.1	+	+	+	+	+	+/-	+/-	+/-
slc25a4	PEO3, PEO2, ANT1, T1	BC072091.1	+	+	+	+	+	-	-	-
slc25a5	ANT2, T2, 2F1, T3	BC043821.1	+	+	+	+	+	-	-	-
slc25a10	DIC	BC070665.1	-	++	-	-	-	-	-	-
slc25a11	SLC20A4,OGC	BC072308.1	+	+	+	++	++	+	+	+
slc25a20	CACT, CAC	BC043827.1	++	++	++	+	+	+	+	+
slc25a22	GC1, FLJ13044	BC063272.1	+	+	+	-	-	+/-	+/-	-
slc25a32	MFTC	BC087370.1	+	+	+	+	+	+	+	-
slc25a39	FLJ22407, CGI-69	BC073249.1	+/-	+/-	+/-	+/-	+/-	-	-	-
slc25a44	FLJ90431, KIAA0446	BC076803.1	++	++	+	+	+	+	+	-
slc26a1	SAT-1, EDM4	BU904894.1	++	++	++	-	-	-	-	-
slc26a6	-	BC075145.1	++	++	++	-	-	-	-	-
slc26a11	-	CA988173.1	-	++	-	-	-	-	-	-
slc27a7	-	BX850807.1	+/-	+/-	-	-	+	+	+/-	-
slc28a1	CNT1	CD098603.1	++	++	++	-	-	-	-	-
slc29a3	ENT3, FLJ11160	BC077451.1	++	++	-	-	-	-	-	-
slc30a7	ZnTL2, ZNT7	BC070769.1	+/-	++	++	-	-	-	-	-
slc30a8	-	BG037315.1	+/-	+/-	+/-	+/-	+/-	-	-	++
slc30a9	C4orf1, HUEL, ZNT9, GAC63	BC078104.1	++	++	+	++	+	+/-	+/-	-
slc31a1	COPT1, hCTR1, CTR1	BC075178.1	++	++	++	++	+	+	+	+

Additional data file 2: Summary of marker gene expression domains for the *Xenopus* stage 35/36 pronephros

Gene	Synonyms	GenBank acc. no.	PT1	PT2	PT3	IT1	IT2	DT1	DT2	CT
slc31a2	COPT2, hCTR2, CTR2	CA971177.1	++	++	++	-	-	-	-	-
slc33a1	ACATN, AT-1	BC068928.1	++	++	-	-	-	-	-	-
slc34a3	NPTIIc, FLJ38680	BC082530.1	++	++	++	-	-	-	-	-
slc35a1	CMPST, hCST	CA791665.1	++	++	++	-	-	-	-	-
slc35a4	-	EB646007.1	+	+	-	+	+	-	-	-
slc35a5	FLJ20730	BC078070.1	++	++	+	+	-	+	+	+
slc35b2	UGTrel4	BC044702.1	+	+	+	-	-	-	-	-
slc35c1	FUCT1, FLJ11320	BJ039584.1	+	+	+	-	-	-	-	-
slc35f2	FLJ13018	BC084761.1	++	++	++	-	-	-	-	-
slc36a1	LYAAT-1, PAT1, TRAMD3	BC070857.1	++	++	+	-	-	-	-	-
slc37a2	FLJ00171	BC042235.1	-	++	++	-	-	-	-	-
slc38a2	SAT2, ATA2, KIAA1382, SNAT2	BC077990.1	-	-	-	-	+	+	+	+/-
slc38a7	-	BC076791.1	++	++	++	-	-	-	-	-
slc39a6	LIV-1	CA788000.1	-	-	-	-	-	+/-	+/-	+/-
slc39a8	BIGM103	BP686084.2	+	+	-	-	-	-	-	-
rhbg	SLC42A2	BC078079.1	-	+	+	-	-	-	-	-
rhcg	SLC42A3, C15orf6, RHGK, PDRC2	BC084943.1	-	-	-	-	-	-	++	-
slc43a2	MGC34680	BC074223.1	++	++	+	+	+	+	+	+
cldn3	C7orf1, CPETR2,RVP1	BC079722.1	++	++	++	+	+	++	+	-
cldn4	CPETR, CPETR1, CPE-R, WBSCR8, hCPE-R	BC099009.1	-	-	-	+	+	+	+	+
cldn6	-	BC077402.1	++	++	++	+	+	+	+	++
cldn8	-	DR877133.1	-	-	-	-	+	-	-	-
cldn12	-	BC088962.1	++	+	+	+/-	+/-	+/-	+/-	-
cldn14	DFNB29	BC074122.1	-	-	-	++	++	++	-	-
cldn16	PCLN1	CD100665.1	-	-	-	++	++	++	-	-
cldn19	-	BC082674.1	-	-	-	++	++	+/-	+/-	+/-
clcnk	Clcnka, Clcnkb	NM_001085839	-	-	-	++	++	++	++	++
kcnj1	Kir1.1, ROMK1	CF522101.1	-	-	-	++	-	++	++	+
calb1	CALB1	U76636.1	-	-	-	-	-	-	-	+
