# Supplementary material for: Organization of the pronephric kidney revealed by large-scale gene expression mapping
Source: Genome Biol. 2008 May 20;9(5):R84. doi: 10.1186/gb-2008-9-5-r84 (PMC2441470; doi:10.1186/gb-2008-9-5-r84)
Supplement: Additional data file 3 — Presented is a table listing marker genes expressed in the proximal tubule of the stage 35/36 pronephric kidney, as determined by whole-mount in situ hybridization. Genes expressed exclusively in this compartment are indicated with asterisks. [file gb-2008-9-5-r84-S3.pdf]

Additional data file 3: Genes expressed in the proximal tubule of the stage 35/36 pronephric kidney
* Genes expressed exclusively in this compartment

Gene	Synonyms	Gene family	GenBank acc. no.
slc1a1*	EAAC1, EAAT3	The high-affinity glutamate and neutral amino acid transporter family	CV079713.1
slc2a2*	GLUT2	The facilitative glucose transporter family	BC070704.1
slc2a8	GLUTX1, GLUT8	The facilitative glucose transporter family	CB593014.1
slc2a10*	GLUT10	The facilitative glucose transporter family	BC073721.1
slc2a11*	GLUT11	The facilitative glucose transporter family	CF519465.1
slc2a13*	HMIT	The facilitative glucose transporter family	BC092027.1
slc3a1*	CSNU1, D2H, RBAT	The heavy subunits of the heteromeric amino acid transporters	BU903456.1
slc3a2	4T2HC, 4F2, NACAE	The heavy subunits of the heteromeric amino acid transporters	BC042234.1
slc4a2	EPB3L1, AE2, HKB3	The bicarbonate transporter family	BG348033.1
slc4a4	NBC1, HNBC1, NBC2, pNBC, hhNMC, SLC4A5	The bicarbonate transporter family	BU905206.1
slc4a7*	SLC4A6, NBC3, SBC2	The bicarbonate transporter family	BC070701.1
slc4a11	CHED2, dJ794I6.2, BTR1	The bicarbonate transporter family	BU904542.1
slc5a1*	SGLT1, D22S675	The sodium glucose cotransporter family	CA974591.1
slc5a2*	SGLT2	The sodium glucose cotransporter family	BC081106.1
slc5a6*	SMVT	The sodium glucose cotransporter family	BF611525.1
slc5a8	AIT	The sodium glucose cotransporter family	BC060005.1
slc5a9*	SGLT4	The sodium glucose cotransporter family	CA788193.1
slc5a11*	KST1, SMIT2, SGLT6	The sodium glucose cotransporter family	AB008225.1
slc6a13*	GAT2	The sodium- and chloride-dependent neurotransmitter transporter family	BC060418.1
slc6a14	-	The sodium- and chloride-dependent neurotransmitter transporter family	BU911733.1
slc6a19*	-	The sodium- and chloride-dependent neurotransmitter transporter family	BC081075.1
slc7a6	y+LAT-2, KIAA0245, LAT3, LAT-2	The cationic amino acid transporter/ glycoprotein-associated amino-acid transporter family	BQ736312.1
slc7a7*	y+LAT-1	The cationic amino acid transporter/ glycoprotein-associated amino-acid transporter family	BC072040.1
slc7a8*	LPI-PC1, LAT2	The cationic amino acid transporter/ glycoprotein-associated amino-acid transporter family	BC044971.1
slc7a13*	AGT-1, XAT2	The cationic amino acid transporter/ glycoprotein-associated amino-acid transporter family	BC060020.1
slc9a6*	NHE6, KIAA0267	The Na+/H+ exchanger family	CA987997.1
slc12a6	KCC3, ACCPN	The electroneutral cation-Cl cotransporter family	BC054325.1
slc13a3*	NADC3, SDCT2	The human Na+-sulfate/carboxylate cotransporter family	BC075138.1
slc13a5*	NACT	The human Na+-sulfate/carboxylate cotransporter family	BC077435.1
slc15a2*	PEPT2	The proton oligopeptide cotransporter family	BQ386718.1

Additional data file 3: Genes expressed in the proximal tubule of the stage 35/36 pronephric kidney
* Genes expressed exclusively in this compartment

Gene	Synonyms	Gene family	GenBank acc. no.
slc15a4*	PHT1, PTR4	The proton oligopeptide cotransporter family	BC079971.1
slc16a1*	MCT, MCT1	The monocarboxylate transporter family	BC070980.1
slc16a6	MCT6, MCT7	The monocarboxylate transporter family	BC047967.1
slc16a9*	FLJ43803, MCT9	The monocarboxylate transporter family	CF520266.1
slc16a12*	MCT12	The monocarboxylate transporter family	BC074222.1
slc17a5*	SIASD, AST, SD, ISSD, NSD, SIALIN, SLD	The vesicular glutamate transporter family	BI445533.1
slc19a1*	FOLT	The folate/thiamine transporter family	BC073675.1
slco2a1	SLC21A2, PGT, OATP2A1	The organic anion transporting family	BC060473.1
slc22a5*	CDSP, OCTN2, SCD	The organic cation/anion/zwitterion transporter family	BC056014.1
slc22a6*	ROAT1, PAHT, OAT1	The organic cation/anion/zwitterion transporter family	BC081057.1
slc22a13*	ORCTL3, OCTL1, OCTL3	The organic cation/anion/zwitterion transporter family	CB559054.1
slc23a2*	SLC23A1, SVCT2, KIAA0238, YSPL2	The Na+-dependent ascorbic acid transporter family	CF522441.1
slc25a1*	SLC20A3,CTP	The mitochondrial carrier family	BC041303.1
slc25a3	PHC	The mitochondrial carrier family	BC046849.1
slc25a4	PEO3, PEO2, ANT1, T1	The mitochondrial carrier family	BC072091.1
slc25a5	ANT2, T2, 2F1, T3	The mitochondrial carrier family	BC043821.1
slc25a10*	DIC	The mitochondrial carrier family	BC070665.1
slc25a11	SLC20A4,OGC	The mitochondrial carrier family	BC072308.1
slc25a20	CACT, CAC	The mitochondrial carrier family	BC043827.1
slc25a22	GC1, FLJ13044	The mitochondrial carrier family	BC063272.1
slc25a32	MFTC	The mitochondrial carrier family	BC087370.1
slc25a44	FLJ90431, KIAA0446	The mitochondrial carrier family	BC076803.1
slc26a1*	SAT-1, EDM4	The multifunctional anion exchanger family	BU904894.1
slc26a6*	-	The multifunctional anion exchanger family	BC075145.1
slc26a11*	-	The multifunctional anion exchanger family	CA988173.1
slc28a1*	CNT1	The Na+-coupled nucleoside transport family	CD098603.1
slc29a3*	ENT3, FLJ11160	The facilitative nucleoside transporter family	BC077451.1
slc30a7*	ZnTL2, ZNT7	The zinc efflux family	BC070769.1
slc30a9	C4orf1, HUEL, ZNT9, GAC63	The zinc efflux family	BC078104.1
slc31a1	COPT1, hCTR1, CTR1	The copper transporter family	BC075178.1
slc31a2*	COPT2, hCTR2, CTR2	The copper transporter family	CA971177.1
slc33a1*	ACATN, AT-1	The acetyl-CoA transporter family	BC068928.1
slc34a3*	NPTIIc, FLJ38680	The type-II Na+-phosphate cotransporter family	BC082530.1
slc35a1*	CMPST, hCST	The nucleoside-sugar transporter family	CA791665.1
slc35a4	-	The nucleoside-sugar transporter family	EB646007.1
slc35a5	FLJ20730	The nucleoside-sugar transporter family	BC078070.1
slc35b2*	UGTrel4	The nucleoside-sugar transporter family	BC044702.1

Additional data file 3: Genes expressed in the proximal tubule of the stage 35/36 pronephric kidney
* Genes expressed exclusively in this compartment

Gene	Synonyms	Gene family	GenBank acc. no.
slc35c1*	FUCT1, FLJ11320	The nucleoside-sugar transporter family	BJ039584.1
slc35f2*	FLJ13018	The nucleoside-sugar transporter family	BC084761.1
slc36a1*	LYAAT-1, PAT1, TRAMD3	The proton-coupled amino acid transporter family	BC070857.1
slc37a2*	FLJ00171	The sugar-phosphate/phosphate exchanger family	BC042235.1
slc38a7*	-	The system A and N, sodium-coupled neutral amino acid transporter family	BC076791.1
slc39a8*	BIGM103	The metal ion transporter family	BP686084.2
rhbg*	SLC42A2	The Rh ammonium transporter family	BC078079.1
slc43a2	MGC34680	The Na+-independent, system-L-like amino acid transporter family	BC074223.1
cldn3	C7orf1, CPETR2,RVP1	Claudins	BC079722.1
cldn6	-	Claudins	BC077402.1
cldn12	-	Claudins	BC088962.1
