# Supplementary material for: Organization of the pronephric kidney revealed by large-scale gene expression mapping
Source: Genome Biol. 2008 May 20;9(5):R84. doi: 10.1186/gb-2008-9-5-r84 (PMC2441470; doi:10.1186/gb-2008-9-5-r84)
Supplement: Additional data file 4 — Presented is a table listing marker genes expressed in the intermediate tubule of the stage 35/36 pronephric kidney, as determined by whole-mount in situ hybridization. Genes expressed exclusively in this compartment are indicated with asterisks. [file gb-2008-9-5-r84-S4.pdf]

Additional data file 4: Genes expressed in the intermediate tubule of the stage 35/36 pronephric kidney
* Genes expressed exclusively in this compartment

Gene	Synonyms	Gene family	GenBank acc. no.
slc2a4	GLUT4	The facilitative glucose transporter family	BC073012.1
slc4a11	CHED2, dJ794I6.2, BTR1	The bicarbonate transporter family	BU904542.1
slc5a8	AIT	The sodium glucose cotransporter family	BC060005.1
slc6a9*	-	The sodium- and chloride-dependent neurotransmitter transporter family	BQ737355.1
slc6a14	-	The sodium- and chloride-dependent neurotransmitter transporter family	BU911733.1
slc7a6	y+LAT-2, KIAA0245, LAT3, LAT-2	The cationic amino acid transporter/ glycoprotein-associated amino-acid transporter family	BQ736312.1
slc12a1	NKCC2	The electroneutral cation-Cl cotransporter family	CF520237.1
slc12a6	KCC3, ACCPN	The electroneutral cation-Cl cotransporter family	BC054325.1
slc16a6	MCT6, MCT7	The monocarboxylate transporter family	BC047967.1
slc16a7	MCT2	The monocarboxylate transporter family	BJ059209.1
slc19a2	TRMA, THTR1	The folate/thiamine transporter family	BC070848.1
slc20a1*	GLVR1, PiT-1, Glvr-1	The type-III Na+-phosphate cotransporter family	BU903168.1
slco2a1	SLC21A2, PGT, OATP2A1	The organic anion transporting family	BC060473.1
slc25a3	PHC	The mitochondrial carrier family	BC046849.1
slc25a4	PEO3, PEO2, ANT1, T1	The mitochondrial carrier family	BC072091.1
slc25a5	ANT2, T2, 2F1, T3	The mitochondrial carrier family	BC043821.1
slc25a11	SLC20A4,OGC	The mitochondrial carrier family	BC072308.1
slc25a20	CACT, CAC	The mitochondrial carrier family	BC043827.1
slc25a32	MFTC	The mitochondrial carrier family	BC087370.1
slc25a44	FLJ90431, KIAA0446	The mitochondrial carrier family	BC076803.1
slc27a7	-	The fatty acid transport protein family	BX850807.1
slc30a9	C4orf1, HUEL, ZNT9, GAC63	The zinc efflux family	BC078104.1
slc31a1	COPT1, CTR1	The copper transporter family	BC075178.1
slc35a4	-	The nucleoside-sugar transporter family	EB646007.1
slc35a5	FLJ20730	The nucleoside-sugar transporter family	BC078070.1
slc38a2	SAT2, ATA2, KIAA1382, SNAT2	The system A and N, sodium-coupled neutral amino acid transporter family	BC077990.1
slc43a2	MGC34680	The Na+-independent, system-L-like amino acid transporter family	BC074223.1
cldn3	C7orf1, CPETR2,RVP1	Claudins	BC079722.1
cldn4	CPETR, CPETR1, CPE-R, WBSCR8, hCPE-R	Claudins	BC099009.1
cldn6	-	Claudins	BC077402.1
cldn8*	-	Claudins	DR877133.1
cldn16	PCLN1	Claudins	CD100665.1
cldn14	DFNB29	Claudins	BC074122.1
cldn19	-	Claudins	BC082674.1
clcnk	Clcnka, Clcnkb	Chloride channel	NM_001085839
kcnj1	Kir1.1, ROMK1	Inwardly rectifying potassium channels	CF522101.1
