# Supplementary material for: Organization of the pronephric kidney revealed by large-scale gene expression mapping
Source: Genome Biol. 2008 May 20;9(5):R84. doi: 10.1186/gb-2008-9-5-r84 (PMC2441470; doi:10.1186/gb-2008-9-5-r84)
Supplement: Additional data file 6 — Presented is a table listing marker genes expressed in the connecting tubule of the stage 35/36 pronephric kidney, as determined by whole-mount in situ hybridization. Genes expressed exclusively in this compartment are indicated with asterisks. [file gb-2008-9-5-r84-S6.pdf]

Additional data file 6: Genes expressed in the connecting tubule of the stage 35/36 pronephric kidney
* Genes expressed exclusively in this compartment

Gene	Synonyms	Gene family	GenBank acc. no.
slc2a4	GLUT4	The facilitative glucose transporter family	BC073012.1
slc6a14	-	The sodium- and chloride-dependent neurotransmitter transporter family	BU911733.1
slc7a6	y+LAT-2, KIAA0245, LAT3, LAT-2	The cationic amino acid transporter/ glycoprotein-associated amino-acid transporter family	BQ736312.1
slc8a1*	NCX1	The Na+/Ca2+ exchanger family	BG371210.1
slc12a3	-	The electroneutral cation-Cl cotransporter family	CA790325.1
slc16a6	MCT6, MCT7	The monocarboxylate transporter family	BC047967.1
slc16a7	MCT2	The monocarboxylate transporter family	BJ059209.1
slc25a11	SLC20A4,OGC	The mitochondrial carrier family	BC072308.1
slc25a20	CACT, CAC	The mitochondrial carrier family	BC043827.1
slc30a8	-	The zinc efflux family	BG037315.1
slc31a1	COPT1, hCTR1, CTR1	The copper transporter family	BC075178.1
slc35a5	FLJ20730	The nucleoside-sugar transporter family	BC078070.1
slc43a2	MGC34680	The Na+-independent, system-L-like amino acid transporter family	BC074223.1
cldn4	CPETR, CPETR1, CPE-R, WBSCR8, hCPE-R	Claudins	BC099009.1
cldn6	-	Claudins	BC077402.1
clcnk	Clcnka, Clcnkb	Chloride channel	NM_001085839
kcnj1	Kir1.1, ROMK1	Inwardly rectifying potassium channels	CF522101.1
calb1*	CALB	Calbindins	U76636.1
